# Reduction of leucocyte cell surface disulfide bonds during immune activation is dynamic as revealed by a quantitative proteomics workflow (SH-IQ)

**DOI:** 10.1098/rsob.180079

**Published:** 2018-09-19

**Authors:** Monika Stegmann, A. Neil Barclay, Clive Metcalfe

**Affiliations:** 1 The Sir William Dunn School of Pathology, University of Oxford, Oxford, UK; 2 National Institute of Biological Standards and Control, Blanche Lane, South Mimms, UK

**Keywords:** allosteric disulfide bonds, redox regulation, thioredoxin, proteomics, immune regulation, immune receptors

## Abstract

Communication through cell surface receptors is crucial for maintaining immune homeostasis, coordinating the immune response and pathogen clearance. This is dependent on the interaction of cell surface receptors with their ligands and requires functionally active conformational states. Thus, immune cell function can be controlled by modulating the structure of either the receptor or the ligand. Reductive cleavage of labile disulfide bonds can mediate such an allosteric change, resulting in modulation of the immune system by a hitherto little studied mechanism. Identifying proteins with labile disulfide bonds and determining the extent of reduction is crucial in elucidating the functional result of reduction. We describe a mass spectrometry-based method—thiol identification and quantitation (SH-IQ)—to identify, quantify and monitor such reduction of labile disulfide bonds in primary cells during immune activation. These results provide the first insight into the extent and dynamics of labile disulfide bond reduction in leucocyte cell surface proteins upon immune activation. We show that this process is thiol oxidoreductase-dependent and mainly affects activatory (e.g. CD132, SLAMF1) and adhesion (CD44, ICAM1) molecules, suggesting a mechanism to prevent over-activation of the immune system and excessive accumulation of leucocytes at sites of inflammation.

## 1. Introduction

The adaptive immune system provides the host with a powerful defence against invading pathogens and diseases by mounting antibody or cellular responses towards pathogens and infected or malignant cells [1]. However, chronic autoimmune diseases can occur if the immune system fails and recognizes self-antigens as foreign. The interaction of cell surface receptors on lymphocytes with their cognate ligands on interacting cells or soluble mediators (e.g. cytokines, chemokines and growth factors) is crucial in mounting and controlling an appropriate immune response. Ligand engagement results in downstream signalling events which initiate a cellular response such as activation, proliferation or migration to sites of infection. Receptor–ligand interactions are highly specific and require the interacting proteins to be present in defined three-dimensional structures, presenting their complementary binding interfaces. These interactions can be controlled by either modulating protein levels or by post-translationally modifying the proteins. Proteomic analysis using mass spectrometry provides a non-invasive and unbiased tool to quantify relative protein levels, to identify post-translational modifications of amino acid side chains (i.e. phosphorylation [2], acetylation [3] and ubiquitination [4,5]) or to identify cleavage sites and has been used to analyse many different cellular systems. Here, we focus on the control of protein function by redox-mediated cleavage of labile disulfide bonds [6–8]. Disulfide bonds are covalent bonds between the sulfur atoms of two cysteines. Most disulfide bonds are evolutionally conserved through protein families and across species [9], and are essential elements of many structural motifs such as immunoglobulins [10] and epidermal growth factor domains, where they offer mechanical stability to the protein fold in the harsh extracellular environment. However, it is becoming increasingly clear that some disulfide bonds can be post-translationally cleaved in by thiol oxidoreductase enzymes [6]. These labile disulfide bonds are either catalytic (found at the active site of cysteine thiol reductase and thiol isomerase enzymes) or allosteric (found in protein motifs that when reduced mediate an allosteric change in the protein structure modulating protein function). Several blood and plasma proteins contain allosteric disulfide bonds which have been implicated in thrombus formation and homeostasis [11,12]. More recently, proteins on the surface of immune cells have been identified as being redox regulated in several immunological functions (reviewed in [13,14]).

Upon reduction, the highly reactive thiol groups (SH) of the cysteine residues are liberated, which provides a unique chemical handle for selective labelling of proteins that contain them [15] and subsequent enrichment for mass spectrometric analysis. We have previously developed a first-generation, non-quantitative mass spectrometry-based method that identified a vast number of leucocyte cell surface proteins with supposed redox labile disulfide bonds [14]. To identify the proteins with labile disulfide bonds whose function is likely to be affected by reducing conditions, it is crucial to quantify the extent of reduction. The more reduced a protein is, the more likely it is that this manifests in a biological effect. Furthermore, quantitation of labile disulfide bond reduction will allow investigating the reduction kinetics.

Here, we report the development of SH-IQ, a label-free quantitative proteomics workflow for the identification and quantitation of labile disulfide bond reduction. This showed that only a subset of the 86 proteins previously identified to contain labile disulfide bonds [14] are significantly reduced using the chemical reductant TCEP. Furthermore, SH-IQ revealed the dynamic and highly regulated reduction of labile disulfide bonds during immune activation in a mixed lymphocyte reaction (MLR).

## 2. Experimental procedures

### 2.1. PBMC isolation and culture of cells

Human peripheral blood mononuclear cells (PBMCs) were isolated from leucocyte cones of healthy donors by standard gradient centrifugation using Ficoll Plaque Premium (GE healthcare). PBMCs were then harvested from the interface, washed with PBS at 400*g* for 10 min and twice at 200*g* for 20 min. PBMCs were maintained at 37°C in a 5% CO_2_ atmosphere in RPMI 1640 medium, supplemented with 10% FCS, 100 U ml^−1^ penicillin and 100 µg ml^−1^ streptomycin, 2 mM L-glutamine, 1 mM sodium pyruvate and 1% MEM non-essential amino acids and 25 µM of the thiol-oxidoreductase inhibitor PX-12 when indicated. In a MLR, PBMCs isolated from two donors were mixed at a 1 : 1 ratio to a final concentration of 1–2 × 10^6^ cells ml^−1^.

2B4 Saito hybridoma T cells [16] were maintained at 37°C in a 10% CO_2_ atmosphere in DMEM medium, supplemented with 10% FCS and 100 U ml penicillin and 100 µg ml^−1^ streptomycin.

### 2.2. Flow cytometric and flow imaging analysis of cells surface markers and cell surface thiol levels

For flow cytometry, the following antibodies and reagents were used at the indicated dilutions or concentrations: CD69-APC (Invitrogen, MHCD6905, d1/100), TCR *α*/β-PE (Biolegend, 306708, d1/100), TCR *α*/β-biotin (AbD Serotec, MCA1413B, d1/5), streptavidin-FITC (AbD Serotec, STAR2B, d1/200), Alexa-488-maleimide (Thermo Scientific, A-10254, 25 µM), live/dead marker (Thermo Scientific, L10120, d1/250), propidium iodide (Thermo Scientific, P3566, 1 µM).

To analyse expression of cell surface markers and thiol levels, 1 × 10^6^ cells (2B4T cells or PBMCs) were reduced with 2.5 mM tris(2-carboxyethyl)phosphine hydrochloride (Sigma-Aldrich, TCEP) for 15 min when indicated, washed with FACS buffer, stained with 100 µl of the indicated primary antibody or Alexa-488-maleimide in FACS buffer (0.25% BSA and 0.1% NaN_3_ in PBS) for 30 min on ice, washed with 4 ml FACS buffer, incubated with 100 µl of the indicated secondary antibody if required for 30 min and stained with 100 µl of the live/dead marker for another 20 min. Cells were then washed with 4 ml FACS buffer and fixed (2% formaldehyde in FACS buffer). The data were acquired on a FACSCalibur (BD Biosciences) and analysed with the FlowJo X software. Non-viable cells were gated out based on either the uptake of the live/dead label or size and granularity as is indicated for each experiment.

Localization of Alexa-488-maleimide labelling was analysed by imaging flow cytometry using ImageStream (Amnis).

### 2.3. Differential thiol labelling of cell surface proteins for SH-IQ

2B4 T cells (1 × 10^8^ cells) were washed with 50 ml PBS supplemented with 1% BSA (PBS/BSA) and incubated with 7.5 ml 2.5 mM methyl-PEG_12_-maleimide (Thermo Scientific, MPM) for 20 min. After washing the cells twice with 25 ml PBS/BSA to remove excess label, labile disulfide bonds were reduced with 10 ml 2.5 mM TCEP in PBS/BSA for 15 min, washed twice with 25 ml PBS/BSA and labelled with 2.5 ml 2.5 mM maleimide-PEG_2_-biotin (Thermo Scientific, MPB) for 20 min. PBMCs (1 × 10^8^ cells) were directly labelled with 2.5 ml 2.5 mM MPB for 25 min on ice.

### 2.4. Cell lysis and capture of biotinylated plasma membrane proteins

The cells were washed twice with 25 ml PBS/BSA, harvested by centrifugation for 5 min at 500*g* and lysed in 2 ml PBS containing 1% Triton X-100 (TX-100) and 100 µl protease inhibitor cocktail (Sigma-Aldrich) for 20 min on ice. The lysate was then cleared by centrifugation at 15 000*g* for 15 min, the supernatant collected and equivalent amounts of protein were purified for membrane proteins using lentil lectin agarose beads (300 µl slurry was equilibrated with buffer A, i.e. PBS containing 0.1% TX-100). Membrane proteins were allowed to bind for 45 min, the resin washed three times with 5 ml buffer A and glycosylated proteins eluted with 1.5 ml buffer B (buffer A containing 10% α-methyl glucoside) for 45 min. The eluted membrane proteins were further purified for MPB-tagged proteins using monomeric avidin agarose beads; non-reversible biotin binding sites of 350 µl slurry were blocked with 2 ml buffer C (2.5 mM biotin in buffer A) and equilibrated with buffer A. Biotinylated proteins were bound for 45 min, the beads washed four times with 5 ml buffer A and the biotinylated proteins eluted with 1 ml buffer C for 45 min.

### 2.5. Deglycosylation and digestion of maleimide-PEG_2_-biotin-labelled membrane proteins

The enriched biotinylated membrane protein fraction was loaded onto a 10 kDa cut-off filter (Vivacon500, Sartorius), proteins were denatured with 8 M urea, disulfide bonds reduced with 10 mM TCEP, cysteines alkylated with 10 mM iodoacetamide (IAA) and the detergent was washed off with 8 M urea. Proteins were then deglycosylated with 500 units PNGaseF (NEB) over night at 37°C and subsequently digested with 1 µg trypsin (Promega) in 25 mM ammonium bicarbonate over night at 37°C. Peptides were eluted from the filter with 0.1% formic acid followed by 0.1% formic acid in 50% acetonitrile and 0.1% formic acid in 80% acetonitrile. The sample was then dried in a vacuum centrifuge and the tryptic peptides desalted on a C18 column before injecting into an HPLC-coupled mass spectrometer.

### 2.6. Mass spectrometry analysis

Peptides were reconstituted in 0.1% formic acid in 2% acetonitrile and separated on an in-house-packed 25 cm C18 column (75 µm inner diameter column, 3 µm diameter C18 Maisch phase) using an Ultimate 3000 nano HPLC (Dionex) in the direct injection mode to a QExactive mass spectrometer (Thermo). Separation was conducted with a gradient of 5–30% buffer B (0.1% formic acid in acetonitrile) for 90 min, followed by 30%–55% buffer B for 20 min and 98% buffer B for 5 min (buffer A: 0.1% formic acid) at a flow rate of 300 nl min^−1^. All data were acquired in a data-dependent mode, automatically switching from MS to collision-induced dissociation MS/MS on the 20 most abundant ions with a precursor ion scan range of 350–1650 m/z. Charge state 1+ ions were rejected. Full scan MS spectra were acquired at a resolution of 70 000 and MS/MS scans at 17 000 at a target value 3 × 10^6^ and 1 × 10^5^ ions, respectively. Dynamic exclusion was enabled with an exclusion duration of 40 s.

### 2.7. Data analysis

SH-IQ data were analysed using Progenesis QI software (Nonlinear Dynamics) to perform label-free quantitation. MS/MS spectra were searched against the UniProt mouse or human reference proteome using the external search engine Mascot (Matrix Science, Boston, MA). Precursor mass tolerance was set at 10 ppm and a fragment tolerance at 0.02 Da, and the precursor ion charge state to 2+, 3+ and 4+. Variable modifications were defined as deamidation on asparagine (N) and glutamine (Q), oxidation on methionine (M) and alkylation with MPB or IAA on cysteines (C). The enzyme specificity was set to trypsin with a maximum of two missed cleavages. The target-decoy-based false discovery rate for proteins was set to 1%. Peptide precursor intensities were log_2_-transformed and normalized using quantile normalization [17] to minimize any technical variation in the sample preparation (electronic supplementary material, figure S1). For quantitation, the average precursor area from at least two unique peptides per protein with a minimum Mascot ion score of 20 was used and expressed as a relative fold change between TCEP and control. The average fold change (AFC) was then analysed with the linear models for microarray data (Limma) statistical package [18]. Significance was determined by applying a paired *t*-test to the mean fold changes and the probability values (*p*-values) were adjusted for multiple testing by applying a Benjamini–Hochberg correction [19] Fold changes were deemed significant when *p*-values were less than 0.05.

Where indicated, SH-IQ data were analysed using the in-house Central Proteomics Facilities Pipeline (CPFP; http://cpfp-master.molbiol.ox.ac.uk:3001/auth/login [20]) platform, which uses the search engines Mascot, X!Tandem [21] and OMSSA [22] using the parameters indicated previously. Quantitation was carried out using normalized spectral index quantification (SINQ) [23].

## 3. Results

### 3.1. Chemical reduction of 2B4T cells by TCEP is time- and concentration-dependent, and confined to the cell surface

**Figure 1.**
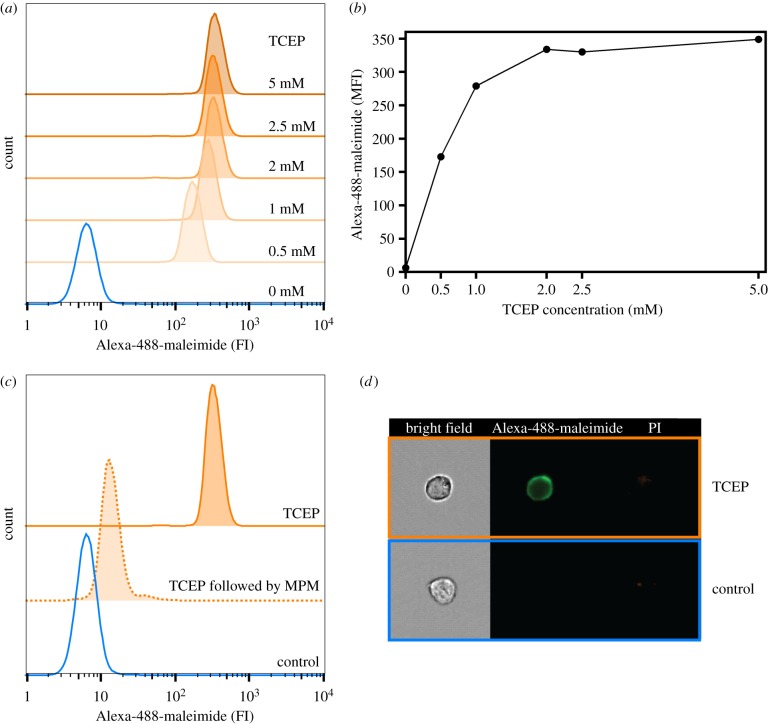
Labile disulfide bonds are reduced in 2B4T cell plasma membrane proteins upon TCEP reduction. 2B4T cells were reduced with the indicated TCEP concentrations for 15 min at room temperature. Free thiols were labelled with Alexa-488-maleimide fluorophore and analysed by flow cytometry. (*a*) Flow cytometry histogram showing 6 populations of cells: control cells (blue) and TCEP reduced cells (orange tones). (*b*) Alexa-488-maleimide median fluorescence intensity (MFI) plotted versus indicated TCEP concentration (from (*a*)). (*c*) 2B4T cells were either incubated with medium (control), reduced with 2.5 mM TCEP (TCEP) or reduced with 2.5 mM TCEP followed by blocking of free thiols with 2.5 mM MPM for 20 min (TCEP followed by MPM) before labelling with Alexa-488-maleimide. (*d*) Flow cytometry image (Amnis ImageStream) of live (propidium iodide negative) control (blue) and TCEP reduced (orange) cells upon visualization of the tagged cysteines.

To develop a quantitative mass spectrometry-based method to analyse the reductive cleavage of labile disulfide bonds, we first established standardized reducing conditions using murine 2B4T cells. The cells were reduced with TCEP and subsequently labelled with the thiol-reactive fluorescent probe Alexa-488-maleimide to visualize the degree and localization of labile disulfide bond reduction. The reducing conditions were optimized to obtain maximal reduction while maintaining high cell viability. Increasing the TCEP concentrations in 15 min reduction from 0 mM to 5 mM showed a concentration-dependent increase of fluorescence, reaching a plateau around 2 mM (figure 1*a*,*b*). To ensure the thiol specificity of Alexa-488-maleimide, TCEP (2.5 mM)-reduced cells were alkylated with the non-fluorescent probe MPM before labelling with Alexa-488-maleimide. This effectively blocked Alexa-488-maleimide labelling, confirming its specificity for free cysteines (figure 1*c*).

Cells were visualized by combined confocal microscopy and flow cytometry to confirm cell-surface-specific labelling of thiols by Alexa-488-maleimide. As expected, non-reduced control cells showed no fluorescence as the probe is unable to penetrate the plasma membrane and label intracellular proteins. TCEP-reduced cells showed cell-surface-specific Alexa-488-maleimide labelling with very little fluorescence occurring from the cytoplasm or nucleus (figure 1*d*). This showed that Alexa-488-maleimide can be used to selectively label and visualize cell surface thiols. Optimal reduction conditions were 2.5 mM TCEP for 15 min.

### 3.2. A tandem lectin-avidin affinity purification enhances the analysis of labile disulfide bond-containing cell surface proteins by mass spectrometry

To accurately quantify the reduction of labile disulfide bonds in plasma membrane proteins, enrichment is essential due to the low relative abundance of plasma membrane proteins compared to intracellular proteins [24], and only a given proportion of a protein population will be affected by the reducing environment. For the most accurate quantitation of protein levels, the abundance and therefore the protein coverage and peptide ion scores need to be increased, without introducing bias, over cell lysate alone. Most cell surface proteins are N-glycosylated and can be enriched using lectin-based purification [25]. By labelling cysteines derived from reduced labile disulfide bonds with a biotinylated probe, an additional avidin affinity purification can be performed to enrich further for labile disulfide bond-containing proteins. To determine the best compromise between loss of material and efficient enrichment of biotinylated cell surface glycoproteins, we compared lectin-based purification of N-glycosylated proteins, avidin-based purification of biotinylated labile disulfide bond-containing plasma membrane proteins and a tandem approach that combines the two.

**Figure 2.**
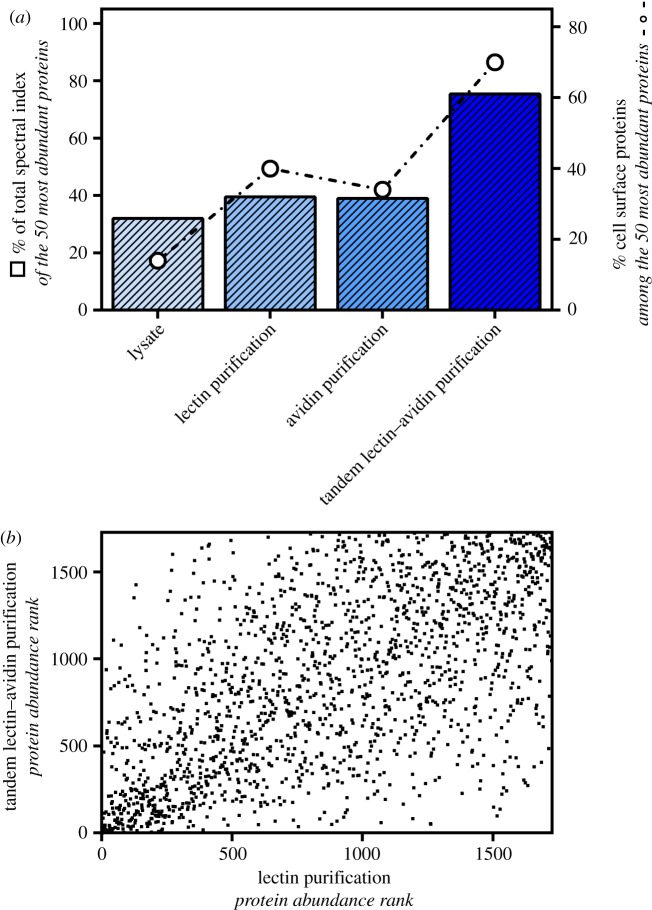
Efficient enrichment of biotinylated cell surface proteins by tandem lectin–avidin purification. (*a*) The percentage coverage of the total spectral index of the 50 most abundant proteins for each purification condition is represented by solid bars (left *y*-axis). The contribution of proteins that can localize to the cell surface is indicated by black circles (right *y*-axis). (*b*) Protein abundance rank (the protein with the highest abundance is assigned rank 1) plot of proteins after lectin versus tandem lectin–avidin purification. There is no linear correlation between the two conditions (*R*^2^ = 0.3811).

2B4 mouse hybridoma T cells were reduced with TCEP and free cysteines alkylated with the biotin probe MPB. The cell lysate was then split into four fractions, processed as indicated (no enrichment, lectin-based enrichment, avidin-based enrichment, tandem lectin-avidin enrichment) and analysed by LC-MS/MS. The measured MS/MS data were searched in the CPFP [20] and relative protein abundances in each fraction were determined with label-free quantitation using the normalized spectral count of proteins determined by SINQ [23]. To determine the degree of enrichment of proteins for each purification method, the combined normalized spectral count of the 50 most abundant proteins identified by each method was compared to total normalized spectral count of all the proteins identified in each sample. The more enrichment there is, the greater the contribution the top 50 identifications will have to the total normalized spectral count when compared to the cell lysate with no enrichment. The top 50 identifications for each purification method are listed in electronic supplementary material, tables S1–S4. Neither lectin nor avidin purification increased the contribution of the 50 most abundant proteins to the total normalized spectral count compared to the whole cell lysate, indicating that the complexity of the samples is comparable to cell lysate (figure 2*a*). However, more than twice as many cell surface proteins were identified as determined by gene ontology analysis, showing that although still complex there is an enrichment of cell surface proteins. For the tandem lectin–avidin purification, the contribution of the 50 most abundant proteins to the total normalized spectral count increases from around 45% to 60%, indicating an efficient enrichment and less complex sample. Importantly, the cell surface protein fraction increases from around 35% to 70% (figure 2*a*) with more unique peptides per protein, increased coverage per protein and increased peptide ion scores, allowing more accurate protein quantitation. Moreover, the data showed that the lectin purification preceding the avidin purification did not introduce any bias which is shown by the poor correlation (*R*^2^ = 0.3811) between the protein abundance after each purification step (figure 2*b*).

### 3.3. SH-IQ: a quantitative, label-free proteomics workflow to assess labile disulfide bond reduction on plasma membrane proteins

**Figure 3.**
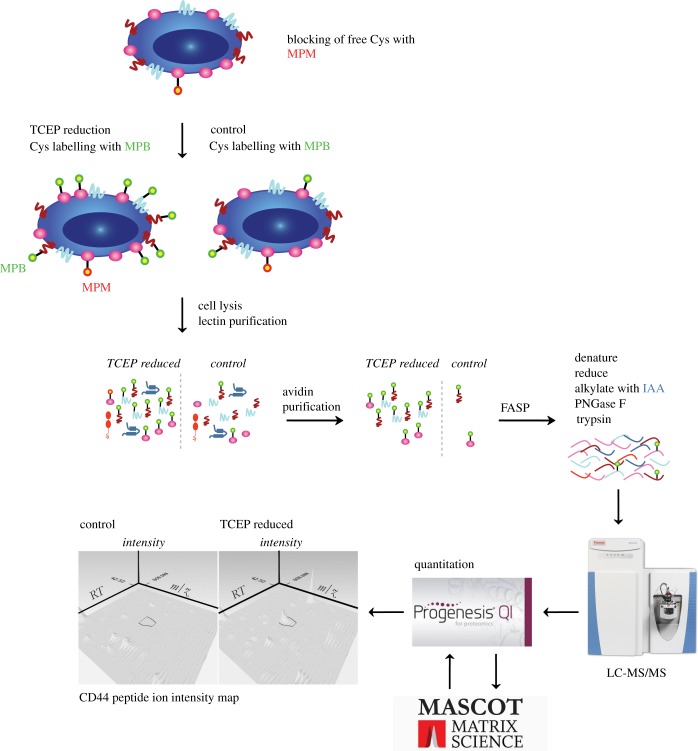
Illustration of the SH-IQ proteomics workflow to quantify labile disulfide bond reduction. Free cysteines were pre-blocked with MPM and cysteines originating from a reduced labile disulfide bond labelled with MPB. Biotinylated glycoproteins were enriched by tandem lectin–avidin purification and the samples prepared for mass spectrometry analysis by filter aided sample preparation (FASP). Protein quantitation was carried out in Progenesis QI. Ion intensity maps from the different runs were aligned, an aggregate matrix was created and searched with Mascot (precursor tolerance 10 ppm, fragment tolerance 0.02 Da, Mascot ion score greater than or equal to 20). This is illustrated on the example of a CD44 peptide in this figure. The data were normalized using quantile normalization and analysed with the statistical package Limma based on at least two unique peptides per protein.

We next performed a proof-of-concept experiment where we identified and quantified (IQ) free cysteine (SH)-containing proteins in a label-free proteomic experiment as a measure of labile disulfide bond reduction (SH-IQ). 2B4 hybridoma T cells were either reduced with TCEP or treated with buffer as a control and free cysteines labelled with MPB. The more labile a disulfide bond is, the more of a given protein population is reduced and labelled with MPB and the more protein is therefore enriched after tandem lectin–avidin purification. The quantitative proteomic workflow SH-IQ is summarized in figure 3.

**Figure 4.**
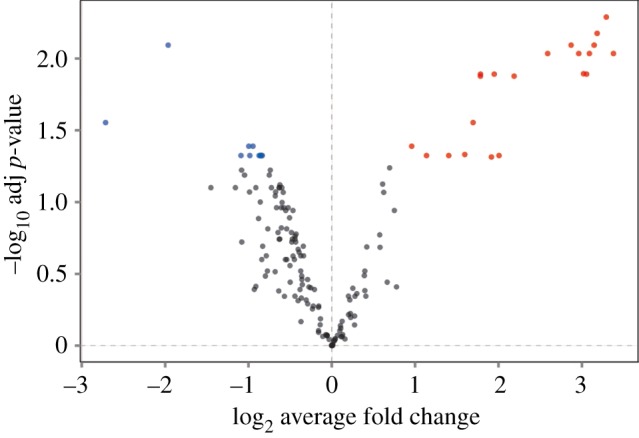
SH-IQ volcano plot. The *x*-axis represents the log_2_ average fold change in TCEP reduced versus control samples and the *y*-axis represents the −log_10_ adjusted *p*-value for each quantified protein. Proteins associated with a *p*-value < 0.05 are coloured in red when the fold change is positive and in blue when negative. Quantitation was carried out on the 163 proteins that were present in all three biological replicates with greater than or equal to 2 unique peptides per protein and a Mascot ion score of greater than or equal to 20.

Upon applying SH-IQ to 2B4T cells, we identified 163 proteins (electronic supplementary material, table S5), including 124 (76%) proteins that are associated with the cell surface as determined by gene ontology (cell surface, plasma membrane, extracellular space, extracellular region, integral component of plasma membrane or external side of plasma membrane). Of these, 32 were found to be differentially abundant (*p*-value < 0.05), with 21 proteins more abundant in the TCEP reduced group (figure 4 and table 1). The higher the TCEP to control ratio, the more labile a disulfide bond in a protein. As expected, proteins with known labile disulfide bonds (CD44 [26], Integrin *β*3 [27–30] and CD30 [31]) were found to be among the proteins with the most labile disulfide bonds (table 1). Furthermore, we identified 16 novel proteins to contain labile disulfide bonds. The nine proteins that showed a decreased abundance are mostly intracellular proteins, suggesting either non-specific binding to the resin or co-purification as protein complexes.

**Table 1.** Quantitation of labile disulfide bond reduction in leucocyte cell surface proteins after TCEP reduction. The listed proteins were identified in three biological replicates and their fold change is associated with a *p*-value that is smaller than 0.05. The data were quantile normalized and the average fold change (AFC) was calculated based on a minimum of two unique peptides per protein. Limma was used to analyse the difference in protein abundance between control and reduced samples. The *p*-values were adjusted for multiple testing applying Benjamini–Hochberg correction. Proteins with known labile disulfide bonds are indicated in italics and it is indicated whether the labile disulfide bond has been previously mapped by Metcalfe *et al.* [14].

accession	gene name	protein description	AFC	log_2_(AFC)	log_2_(average abundance)	*p*-value	adjusted *p*-value	mapped by Metcalfe *et al*. [14]
Q9CYA0	Creld2	cysteine-rich with EGF-like domain protein 2	10.39	3.38	18.34	3.92 × 10^−4^	0.009	no
Q62469	Itga2	integrin alpha-2	9.79	3.29	22.83	3.16 × 10^−5^	0.005	no
A2APM1	Cd44	*CD44 antigen*	9.08	3.18	26.27	8.20 × 10^−5^	0.007	yes
Q9QZF2	Gpc1	Glypican-1	8.86	3.15	18.33	2.23 × 10^−4^	0.008	no
Q80V42	Cpm	Carboxypeptidase M	8.52	3.09	20.26	4.95 × 10^−4^	0.009	no
P35456	Plaur	Urokinase plasminogen activator surface receptor	8.32	3.06	18.96	8.92 × 10^−4^	0.013	no
O54890	Itgb3	*Integrin beta-3*	8.11	3.02	24.92	7.84 × 10^−4^	0.013	yes
Q60846	Tnfrsf8	*Tumour necrosis factor receptor superfamily member 8, CD30*	7.8	2.96	24.22	5.10 × 10^−4^	0.009	no
P09055	Itgb1	Integrin beta-1	7.31	2.87	25.39	1.79 × 10^−4^	0.008	yes
Q6P5F6	Slc39a10	zinc transporter ZIP10	6.02	2.59	23.87	4.21 × 10^−4^	0.009	yes
Q8R2Q8	Bst2	bone marrow stromal antigen 2	4.55	2.19	20.06	1.19 × 10^−3^	0.013	no
Q64151	Sema4c	Semaphorin-4C	4.02	2.01	18.94	7.61 × 10^−3^	0.047	no
Q542I8	Itgb2	integrin beta-2	3.86	1.95	28.2	1.03 × 10^−3^	0.013	yes
Q64697	Ptprcap	protein tyrosine phosphatase receptor type C-associated protein	3.77	1.92	20.23	9.53 × 10^−3^	0.049	yes
E9Q5M7	Itgal	integrin alpha-L	3.44	1.78	28.53	9.61 × 10^−4^	0.013	yes
O89001	Cpd	carboxypeptidase D	3.44	1.78	20.28	1.22 × 10^−3^	0.013	no
O35598	Adam10	disintegrin and metalloproteinase domain-containing protein 10	3.24	1.7	21.76	2.75 × 10^−3^	0.028	yes
P01831	Thy1	Thy-1 membrane glycoprotein	3.02	1.6	22.73	6.01 × 10^−3^	0.047	yes
Q3TB92	Milr1	Allergin-1	2.64	1.4	23.52	7.67 × 10^−3^	0.047	no
O09126	Sema4d	Semaphorin-4D	2.2	1.14	24.23	7.58 × 10^−3^	0.047	yes
A2AN91	Susd1	Protein Susd1	1.94	0.96	21.26	4.86 × 10^−3^	0.041	no
Q61753	Phgdh	D-3-phosphoglycerate dehydrogenase	0.56	−0.83	23.44	8.68 × 10^−3^	0.047	no
P97370	Atp1b3	sodium/potassium-transporting ATPase subunit beta-3	0.56	−0.84	24.93	8.00 × 10^−3^	0.047	no
Q03265	Atp5a1	ATP synthase subunit alpha, mitochondrial	0.56	−0.85	24.6	6.68 × 10^−3^	0.047	no
D3YU17	Ncln	Nicalin	0.55	−0.86	20.32	8.84 × 10^−3^	0.047	no
Q8K297	Colgalt1	procollagen galactosyltransferase 1	0.55	−0.87	21.53	9.02 × 10^−3^	0.047	no
Q9JKR6	Hyou1	hypoxia up-regulated protein 1	0.52	−0.95	23.81	4.70 × 10^−3^	0.041	no
P56480	Atp5b	ATP synthase subunit beta, mitochondrial	0.51	−0.98	25.99	9.00 × 10^−3^	0.047	no
Q01965	Ly9	T-lymphocyte surface antigen Ly-9	0.5	−1	25.36	5.01 × 10^−3^	0.041	yes
Q9JLF6	Tgm1	Protein-glutamine gamma-glutamyltransferase K	0.47	−1.09	17.7	7.75 × 10^−3^	0.047	no
P38647	Hspa9	stress-70 protein, mitochondrial	0.26	−1.96	28.28	2.48 × 10^−4^	0.008	yes
Q9CPN9	2210010C04Rik	protein 2210010C04Rik	0.15	−2.71	27.18	2.91 × 10^−3^	0.028	no

### 3.4. Human peripheral blood mononuclear cells show an increase in cell surface thiol levels during a mixed leucocyte reaction

**Figure 5.**
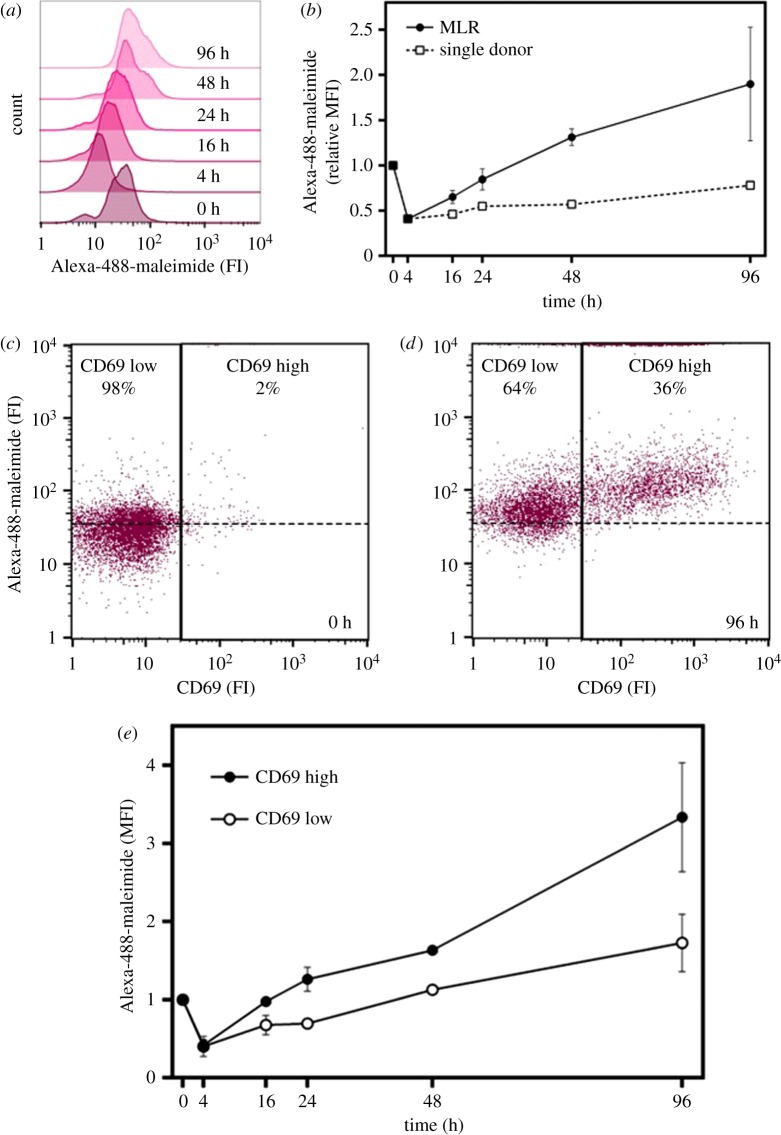
Thiol levels increase on the leucocyte cell surface during immune activation. PBMCs of two donors were mixed at a 1 : 1 ratio and co-cultured for 96 h. (*a*) Cells were removed at the time points indicated for flow cytometry analysis and thiols visualized by Alexa-488-maleimide staining. (*b*) Quantitative analysis of flow cytometry data shown in (*a*). Values of four biological replicates relative to time point 0 h ± standard deviation. Example raw flow cytometry data gated on T cells (expressing α/β TCR chain) showing the increase of thiol levels (Alexa-488-maleimide) correlates to T cell activation (increase in CD69 expression) (*c*) at time 0 and (*d*) after 96 h. (*e*) Quantitative analysis of flow cytometry data at time points taken throughout the MLR. Normalized mean values relative to time point 0 h for three replicates ± s.e. of the mean.

In order to determine the effectiveness of the SH-IQ technology in more physiologically relevant applications, we decided to study cell surface proteins reduced during immune activation in a mixed leucocyte reaction (MLR) [32] with the intent of gaining an insight into the possible reduction mechanisms employed by the cell. Firstly however, labile disulfide bond reduction in the MLR was probed using flow cytometry. Human PBMCs from two donors were co-cultured in the presence of IL-2 to facilitate allogeneic T cell activation and proliferation. PBMC cell surface thiol levels were followed by flow cytometry as described above. Thiol levels increased in a time-dependent manner which indicates labile disulfide bond reduction (figure 5*a*). This increase in cell surface thiols is greater in an MLR relative to an autologous reaction (figure 5*b*). Although the general trend is a time-dependent increase in cell surface thiols, freshly isolated PBMCs showed higher thiol levels compared with 4 h into the MLR, as was previously shown [33], and has been attributed to a phenomenon in the isolation process. These data suggest that reduction of labile disulfide bonds increases as the MLR progresses and the leucocytes become more activated.

To determine whether cell surface disulfide bond reduction on T cells can be correlated to their activation state, levels of the T cell activation marker CD69 were monitored throughout the MLR from time 0 h (figure 5*c*) to 96 h (figure 5*d*) using flow cytometry. Quantitative analysis of the flow cytometry data showed a greater time-dependent increase of cell surface thiols in activated T cells (CD69 high) than in non-activated T cells (figure 5*e*).

### 3.5. Labile disulfide bonds in plasma membrane proteins of activated T cells are cleaved by thiol oxidoreductases

The reduction of labile disulfide bonds on the cell surface of T cells increases as the cells become activated. One hypothesis for the mechanism of reduction is that activated T cells secrete thiol oxidoreductase or isomerase enzymes such as Trx1, PDI, ERp5 and ERp57, which reduce labile disulfide bonds on the cell plasma membrane in a self-regulatory manner.

**Figure 6.**
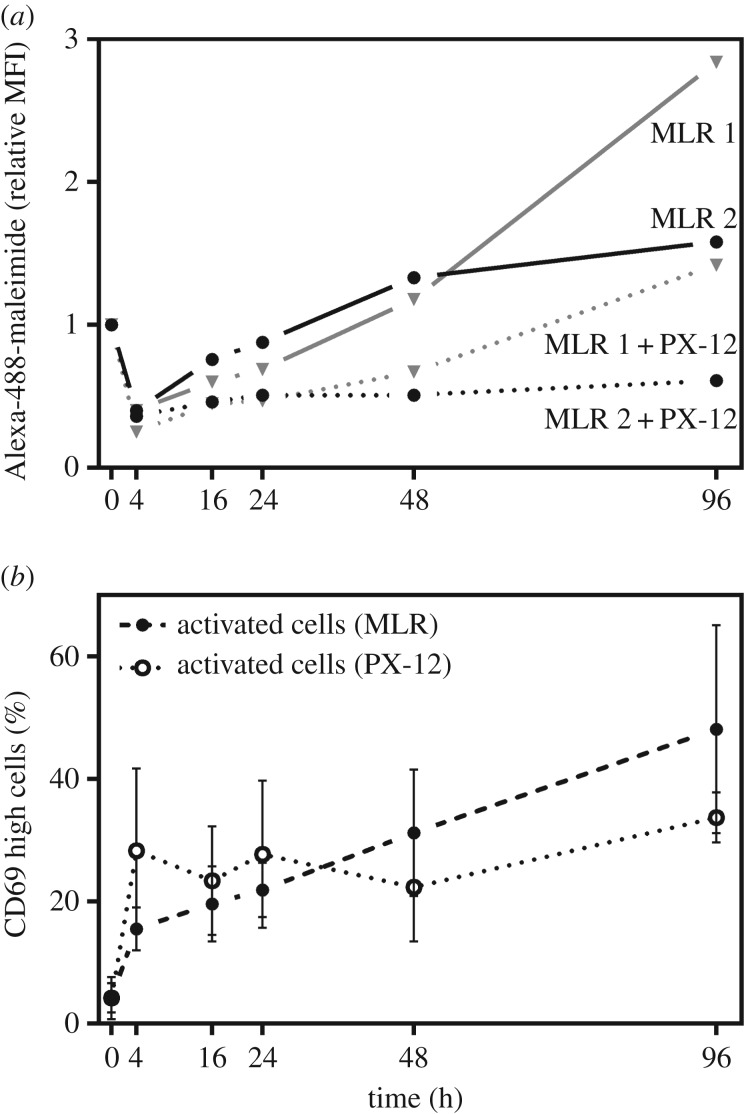
Thioredoxin inhibition affects labile disulfide bond reduction but not T cell activation during immune response. (*a*) MLRs were set up as described in figure 5 and in the presence of the thioredoxin inhibitor PX-12, and analysed by flow cytometry at the time points indicated. Thiol levels (Alexa-488-maleimide staining of T cells, expressing *α*/*β* TCR chain) were followed. The data are from two biological replicates. (*b*) T cell activation state (CD69 expression) throughout the MLR for both the control and PX-12-treated samples.

To test this, MLRs were set up and the mixed cells were split into two, and to one set of cells the irreversible thiol oxidoreductase inhibitor PX-12 [34] was added, which subsequently inhibited the cleavage of labile disulfide bonds in plasma membrane proteins (figure 6*a*) relative to the sample with no inhibitor. This suggests oxidoreductase-mediated labile disulfide bond cleavage is contributing to the increase in free thiols during immune activation. Importantly, PX-12 did not inhibit activation of the T cells, CD69 levels were comparable throughout the MLR in both the control and PX-12-treated samples (figure 6*b*).

### 3.6. SH-IQ reveals dynamic reduction of labile disulfide bonds in PBMC plasma membrane proteins during immune activation

**Figure 7.**
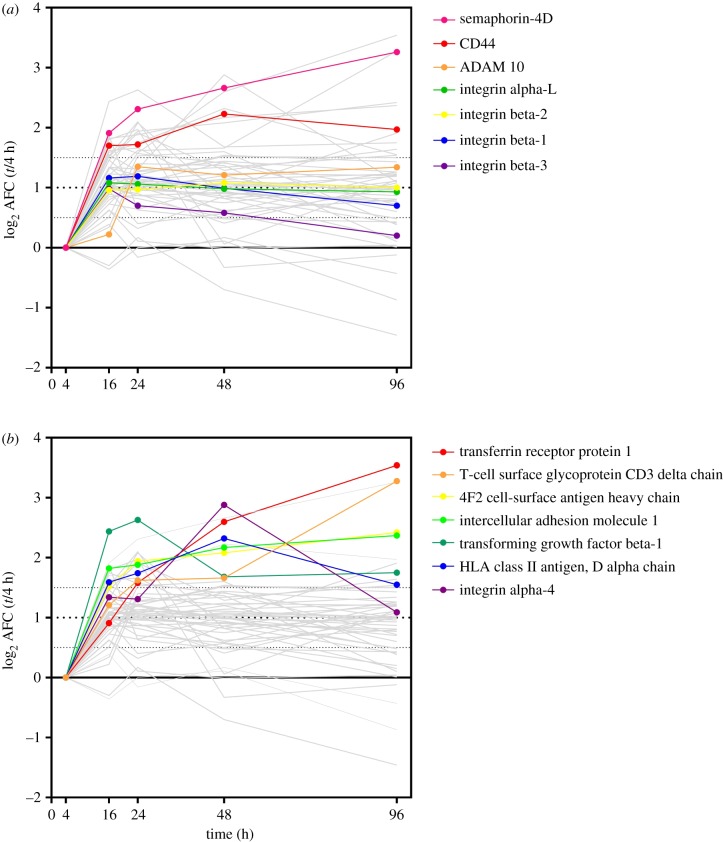
SH-IQ reveals dynamic reduction of labile disulfide bonds during immune activation. The SH-IQ log_2_ average fold change (AFC) of cell surface proteins from two MLRs is plotted versus time. (*a*) Proteins whose reduction was also quantified in this work using 2B4T cells and the chemical reductant TCEP and (*b*) proteins that were previously identified to contain labile disulfide bonds.

To identify and quantify the amount of labile disulfide bond reduction in plasma membrane proteins during immune activation, we applied SH-IQ to the MLR, which was set up as described above. PBMCs removed at the time points indicated, thiols labelled with MPB and the samples processed by following the SH-IQ workflow (figure 3). The AFCs, which are indicative of disulfide bond cleavage, were measured over the course of the MLR (at 0, 4, 16, 24, 48 and 98 h) and are expressed relative to four hours where the thiol levels are the lowest. We identified a total of 53 cell surface proteins across two MLRs with an AFC greater than 2 and revealed for the first time that the reduction of plasma membrane proteins is a dynamic process (figure 7). Proteins with the highest fold changes observed over the course of the MLR, indicating that they were reduced the most, are also proteins that have been previously identified on TCEP-reduced cells, either in this study (figure 7*a* and table 1) or in a previous study (figure 7*b*) [14]. For instance, CD44 [26] which contains a well-characterized allosteric disulfide bond, was rapidly reduced upon immune activation (figure 7*a*), as were other proteins such as semaphorin-4D, transferrin receptor and 4F2 cell-surface antigen heavy chain (figure 7*b*). Furthermore, this study revealed plasma membrane proteins with hitherto unknown and thus uncharacterized labile disulfide bonds that were reduced during immune activation (i.e. CD3 delta chain and TGF beta-1).

Overall, these data demonstrated that the quantitative SH-IQ technology allows the study of the dynamics of labile disulfide bond reduction on plasma membrane proteins in primary cells.

## 4. Discussion

The role of labile or allosteric disulfide bonds in biology is a growing area of interest as it provides a way of modulating biological function. Therefore, controlling this modulation provides a potential means of therapeutic intervention of many diseases, such as those involving the immune system [13], haemostasis [12] and cancer [35]. An increase in free thiols on immune cells upon activation was first described 20 years ago by Lawrence *et al.* [33] and was henceforth confirmed in various studies [36–38]. However, these studies are limited as they only provide global data and do not identify the modified proteins, nor do they provide a quantitative determination of the level of reduction of each protein. A mass spectrometry-based approach developed previously in the laboratory offered significant improvement, as identification of modified proteins became possible [14]; however, this method did not address the quantitation problem. Development of SH-IQ now provides the means for quantitation, which is demonstrated in both a model system using TCEP reduced 2B4T cells and a more physiological setting of an MLR. Thirty-two proteins were identified and quantified in the MLR of which 21 showed AFC greater than twofold. Many of these proteins are implicated in T cell activation (SLAMF1, CD44, CD132, ICAM-1, integrin *β*). However, as it is predominantly CD4^+^ T cells that are activated and undergo proliferation in the MLR, T cell proteins are over-represented in the sample relative to proteins from other cell types. Nonetheless, this provides further evidence that redox regulation of cell surface labile disulfide bonds plays an important role in regulating T cell activation.

To understand the mechanism and implications of labile disulfide bond reduction during immune activation, it is essential to elucidate the effect on individual proteins and how different cell types are affected. Monitoring labile disulfide bond reduction using flow cytometry facilitated to study the effect in relation to T cell activation. Over the course of the MLR, activated T cells undergo more cell surface protein reduction than resting T cells (figure 5*b*). This process is inhibited by PX-12, which was developed as a selective Trx1 inhibitor [34,39]; however, as other oxidoreductases, such as PDI, ERp5 and ERp57, contain thioredoxin-like domains, it is possible that they are also inhibited. Similar effects were observed by Lawrence *et al.* [33] using PMA (independent of antigen presentation) to stimulate PBMCs and the ubiquitous thiol isomerase inhibitor bacitracin. Although it was previously shown that Trx1 and other oxidoreductases are upregulated and secreted from T cells upon activation [38,40], we provide the first global identification of the proteins containing labile disulfide bonds sensitive to oxidoreductases on the surface of T cells undergoing activation and expansion. The self-modulation of cell surface receptors by labile disulfide bond reduction is a concept analogous to the autocrine function of cytokines. Trx1 has previously been implicated as having a cytokine-like role in T cell function; however, the mechanisms underlying the process were never fully elucidated [41].

Two receptors identified by SH-IQ (CD132 [42] and CD44 [26]) have been followed up with functional experiments to investigate the effect of labile disulfide bond reduction on protein function, which were found to be allosteric. Like many proteins identified in the SH-IQ screens, they are associated with the activation of T cells. This adds further evidence to the hypothesis that T cells use labile disulfide bond reduction to regulate key activating receptors on their surface, analogous in action to blocking antibodies such as anti-CD28 which can tune the T cell response by inhibiting the CD28-CD80 interaction [32]. Redox-mediated changes in cell adhesion may also play an important role in controlling activation. ICAM-1 and its interaction partner LFA-1 (integrin α_L_β_2_) were both identified on 2B4T cells and PBMCs using SH-IQ as well as in our previous screen [14]. ICAM-1/LFA-1 is important for lymphocyte adhesion [43] and acts as a co-stimulatory interaction in T cell activation [44]. The CD3 delta chain was identified by SH-IQ as having a labile disulfide bond that becomes reduced as the MLR progressed. All three of the CD3 delta chains, epsilon, delta and gamma, which make up the heterodimeric ectodomains of the TCR contain a conserved CXXC motif in their membrane proximal stalk. These cysteines are essential for TCR-mediated T cell activation and form a disulfide bond which needs to be oxidized for signalling to occur [45]. Although it was found that these disulfide bonds were resistant to Trx1-mediated reduction, CXXC motifs are usually highly strained and labile, so it could be that in the MLR they are exposed upon binding to peptide MHC thus making them accessible to oxidoreductases. In addition, CD3 delta contains a further disulfide bond at the heart of its Ig like domain; however, our previous study showed that disulfide bonds in Ig domains are not usually labile [14]. Platelets require a PDI-mediated disulfide bond exchange to activate integrins *α*_IIb_*β*_III_ [46] and *α*_II_*β*_I_ [47] to allow platelets to adhere to fibrinogen and collagen. Similarly, neutrophils require PDI-activated integrin *α*_M_*β*_II_ for recruitment to sites of vascular inflammation [48].

Another important consideration is what makes the disulfide bond reduction transient. This could be due to two mechanisms: either the disulfide bonds are re-forming or the reduced proteins are being turned over and thus removed from the cell surface. The levels of reduced cell surface proteins generally remain high throughout the MLR suggesting that reduced proteins are not rapidly removed from the cell surface but stay there in the reduced conformation.

Finally, it is becoming increasingly evident that the enzymatic reduction of labile disulfide bonds is an important mechanism by which leucocytes post-translationally control the function of their cell surface receptors. SH-IQ provides a tool to not only identify proteins containing such functional entities but also quantify the relative amount of reduction. For the first time, this has allowed us to study the dynamics of labile disulfide bond reduction throughout the course of an immune reaction. Future application of SH-IQ to patient tissues as well as animal models will be invaluable in identifying potential targets that could be modulated though the development of novel redox therapeutics as well as furthering our general understanding of the immunological role of cell surface redox regulation.

## Supplementary Material

Supplementary tables and figures

## Data accessibility

The datasets supporting this article have been uploaded as part of the electronic supplementary material.
