# Supplementary material for: Reduction of leucocyte cell surface disulfide bonds during immune activation is dynamic as revealed by a quantitative proteomics workflow (SH-IQ)
Source: Open Biol. 2018 Sep 19;8(9):180079. doi: 10.1098/rsob.180079 (PMC6170505; doi:10.1098/rsob.180079)
Supplement: Supplementary tables and figures [file rsob180079supp1.docx]

**Supplementary materials**

**Supplementary Table 1**: **50 most abundant proteins identified from leukocyte whole cell lysate.**

Samples were prepared for mass spectrometry analysis and the results searched using the CPF Pipeline (10 ppm precursor tolerance, 0.02 Da fragment tolerance, 2 missed cleavages). SINQ (FDR 1%) analysis was performed to determine relative protein abundances. For the cell surface proteins listed in the table the following information is shown: Abundance rank (1 indicating the most abundant protein in the sample), UniProt accession number, gene name, Mascot score, emPAI (exponentially modified protein abundance index), molecular weight of the protein in Da, sequence coverage and unique peptide sequences identified.

| **Abundance Rank** | **Accession** | **Gene** | **Description** | **Mascot Score** | **emPAI** | **Mass (Da)** | **Sequence Coverage (%)** | **Unique peptides** |
| --- | --- | --- | --- | --- | --- | --- | --- | --- |
| 1 | P17742 | Ppia | Peptidyl-prolyl cis-trans isomerase A | 878 | 62.49 | 18131 | 65 | 9 |
| 2 | S4R1W1 | Gm3839 | Glyceraldehyde-3-phosphate dehydrogenase | 219 | 4.77 | 18776 | 52 | 7 |
| 3 | P18760 | Cfl1 | Cofilin-1 | 219 | 4.77 | 18776 | 52 | 7 |
| 4 | P06151 | Ldha | L-lactate dehydrogenase A chain | 584 | 4.89 | 40075 | 42 | 12 |
| 5 | P35700 | Prdx1 | Peroxiredoxin-1 | 283 | 7.17 | 22390 | 49 | 8 |
| 6 | P26350 | Ptma | Prothymosin alpha | 793 | 20.11 | 12247 | 23 | 6 |
| 7 | P10126 | Eef1a1 | Elongation factor 1-alpha 1 | 709 | 2.74 | 50424 | 36 | 11 |
| 8 | P14131 | Rps16 | 40S ribosomal protein S16 | 189 | 3.12 | 16549 | 38 | 6 |
| 9 | P58252 | Eef2 | Elongation factor 2 | 1394 | 6.99 | 96222 | 5 | 35 |
| 10 | P62962 | Pfn1 | Profilin-1 | 458 | 29.22 | 15119 | 72 | 8 |
| 11 | O55142 | Rpl35a | 60S ribosomal protein L35a | 458 | 29.22 | 15119 | 72 | 8 |
| 12 | P63017 | Hspa8 | Heat shock cognate 71 kDa protein | 1623 | 9.41 | 71055 | 49 | 25 |
| 13 | P63038 | Hspd1 | 60 kDa heat shock protein, mitochondrial | 1462 | 13.11 | 61088 | 68 | 27 |
| 14 | P62908 | Rps3 | 40S ribosomal protein S3 | 355 | 12.96 | 26828 | 65 | 14 |
| 15 | P14148 | Rpl7 | 60S ribosomal protein L7 | 242 | 2.33 | 31457 | 27 | 8 |
| 16 | P17182 | Eno1 | Alpha-enolase | 1082 | 8.95 | 47453 | 56 | 20 |
| 17 | P34884 | Mif | Macrophage migration inhibitory factor | 79 | 1.09 | 12667 | 17 | 2 |
| 18 | Q6ZWN5 | Rps9 | 40S ribosomal protein S9 | 133 | 4.28 | 22635 | 39 | 8 |
| 19 | P61255 | Rpl26 | 60S ribosomal protein L26 | 60 | 1.29 | 17020 | 17 | 3 |
| 20 | P62270 | Rps18 | 40S ribosomal protein S18 | 299 | 17.29 | 17697 | 57 | 11 |
| 21 | P41105 | Rpl28 | 60S ribosomal protein L28 | 166 | 2.28 | 15781 | 23 | 4 |
| 22 | P10853 | Hist1h2bf | Histone H2B type 1-F/J/L | 472 | 9.54 | 14915 | 42 | 6 |
| 23 | Q03265 | Atp5a1 | ATP synthase subunit alpha, mitochondrial | 1118 | 8.24 | 59830 | 5 | 24 |
| 24 | P11499 | Hsp90ab1 | Heat shock protein HSP 90-beta | 1924 | 9.32 | 83571 | 51 | 18 |
| 25 | P61358 | Rpl27 | 60S ribosomal protein L27 | 163 | 4.94 | 15788 | 33 | 5 |
| 26 | P99027 | Rplp2 | 60S acidic ribosomal protein P2 | 402 | 23.31 | 11644 | 81 | 6 |
| 27 | Q91V55 | Rps5 | 40S ribosomal protein S5 | 268 | 2.95 | 20572 | 37 | 5 |
| 28 | Q64433 | Hspe1 | 10 kDa heat shock protein, mitochondrial | 194 | 4.42 | 10956 | 37 | 4 |
| 29 | P62082 | Rps7 | 40S ribosomal protein S7 | 163 | 1.9 | 22167 | 60 | 5 |
| 30 | Q6ZWZ6 | Rps12 | 40S ribosomal protein S12 | 111 | 1.57 | 14905 | 32 | 3 |
| 31 | P62259 | Ywhae | 14-3-3 protein epsilon | 520 | 8.56 | 29326 | 53 | 11 |
| 32 | P47963 | Rpl13 | 60S ribosomal protein L13 | 199 | 2.2 | 24348 | 26 | 6 |
| 33 | P60867 | Rps20 | 40S ribosomal protein S20 | 107 | 1 | 13478 | 19 | 2 |
| 34 | Q9D1R9 | Rpl34 | 60S ribosomal protein L34 | 86 | 2.97 | 13513 | 28 | 4 |
| 35 | P14211 | Calr | Calreticulin | 233 | 1.43 | 48136 | 27 | 8 |
| 36 | P97351 | Rps3a | 40S ribosomal protein S3a | 456 | 9.56 | 30094 | 49 | 13 |
| 37 | Q9CZN7 | Shmt2 | Serine hydroxymethyltransferase | 687 | 5.4 | 56237 | 45 | 18 |
| 38 | P62702 | Rps4x | 40S ribosomal protein S4, X isoform | 503 | 8.17 | 29807 | 42 | 10 |
| 39 | P51881 | Slc25a5 | ADP/ATP translocase 2 | 318 | 4.53 | 33138 | 41 | 6 |
| 40 | P62900 | Rpl31 | 60S ribosomal protein L31 | 84 | 0.87 | 14987 | 17 | 2 |
| 41 | P62204 | Calm1 | Calmodulin | 275 | 2.69 | 21603 | 34 | 6 |
| 42 | P62806 | Hist1h4a | Histone H4 | 170 | 4.16 | 11360 | 43 | 4 |
| 43 | P63101 | Ywhaz | 14-3-3 protein zeta/delta | 407 | 7.98 | 27925 | 5 | 8 |
| 44 | P84099 | Rpl19 | 60S ribosomal protein L19 | 192 | 1.24 | 23347 | 19 | 4 |
| 45 | P62852 | Rps25 | 40S ribosomal protein S25 | 127 | 1.77 | 13791 | 16 | 3 |
| 46 | P17918 | Pcna | Proliferating cell nuclear antigen | 327 | 2.67 | 29108 | 34 | 7 |
| 47 | P62245 | Rps15a | 40S ribosomal protein S15a | 87 | 2.08 | 12417 | 22 | 3 |
| 48 | P62751 | Rpl23a | 60S ribosomal protein L23a | 178 | 1.22 | 17684 | 21 | 3 |
| 49 | P25444 | Rps2 | 40S ribosomal protein S2 | 423 | 5.05 | 31497 | 38 | 10 |
| 50 | P62830 | Rpl23 | 60S ribosomal protein L23 | 269 | 5.51 | 14970 | S38 | 4 |

**Supplementary Table 2.** **50 most abundant proteins identified after lectin purification.**

2B4 T cells were reduced with TCEP and free cysteines labelled with MPB. The sample was then enriched for glycosylated proteins with lectin beads and prepared for mass spectrometry analysis. The results were searched using the CPF Pipeline (10 ppm precursor tolerance, 0.02 Da fragment tolerance, 2 missed cleavages). SINQ (FDR 1%) analysis was performed to determine relative protein abundances. For the cell surface proteins listed in the table the following information is shown: Abundance rank (1 indicating the most abundant protein in the sample), UniProt accession number, gene name, Mascot score, emPAI (exponentially modified protein abundance index), molecular weight of the protein in Da, sequence coverage and unique peptide sequences identified.

| **Abundance Rank** | **Protein** | **Gene** | **Description** | **Mascot Score** | **emPAI** | **Mass (Da)** | **Sequence Coverage (%)** | **Unique peptides** |
| --- | --- | --- | --- | --- | --- | --- | --- | --- |
| 1 | P01831 | Thy1 | Thy-1 membrane glycoprotein | 1039 | 8.22 | 18297 | 55 | 9 |
| 2 | P06800 | Ptprc | Receptor-type tyrosine-protein phosphatase C | 7279 | 8.39 | 130199 | 54 | 59 |
| 3 | P10404 | Hspa9 | MLV-related proviral Env polyprotein | 4015 | 4.82 | 70709 | 48 | 19 |
| 4 | P10126 | Eef1a1 | Elongation factor 1-alpha 1 | 2325 | 2.3 | 50424 | 39 | 12 |
| 5 | S4R1W1 | Gm3839 | Glyceraldehyde-3-phosphate dehydrogenase | 1668 | 8.17 | 38914 | 51 | 15 |
| 6 | P38647 | Hspa9 | Stress-70 protein, mitochondrial | 4770 | 11.96 | 73701 | 58 | 35 |
| 7 | Q62351 | Tfrc | Transferrin receptor protein 1 | 2882 | 5.61 | 86076 | 4 | 29 |
| 8 | P26350 | Ptma | Prothymosin alpha | 1005 | 9.93 | 12247 | 23 | 6 |
| 9 | P99027 | Rplp2 | 60S acidic ribosomal protein P2 | 552 | 11.19 | 11644 | 92 | 8 |
| 10 | P51881 | Slc25a5 | ADP/ATP translocase 2 | 542 | 2.46 | 33138 | 29 | 5 |
| 11 | P18760 | Cfl1 | Cofilin-1 | 326 | 1.1 | 25074 | 27 | 5 |
| 12 | P63017 | Hspa8 | Heat shock cognate 71 kDa protein | 2595 | 6.94 | 71055 | 5 | 24 |
| 13 | P24668 | M6pr | Cation-dependent mannose-6-phosphate receptor | 516 | 2.67 | 31666 | 4 | 9 |
| 14 | P48962 | Slc25a4 | ADP/ATP translocase 1 | 351 | 2.1 | 33111 | 32 | 6 |
| 15 | P20029 | Hspa5 | 78 kDa glucose-regulated protein | 2923 | 5.87 | 72492 | 46 | 27 |
| 16 | Q8VEM8 | Slc25a3 | Phosphate carrier protein, mitochondrial | 588 | 2.07 | 40167 | 26 | 9 |
| 17 | P14131 | Rps16 | 40S ribosomal protein S16 | 199 | 3.76 | 16549 | 42 | 6 |
| 18 | P62962 | Pfn1 | Profilin-1 | 566 | 7.96 | 15119 | 63 | 7 |
| 19 | E9Q5M7 | Itgal | Integrin alpha-L | 2967 | 2.73 | 129487 | 44 | 37 |
| 20 | P17742 | Ppia | Peptidyl-prolyl cis-trans isomerase A | 775 | 5.29 | 18131 | 61 | 8 |
| 21 | P35980 | Rpl18 | 60S ribosomal protein L18 | 216 | 0.99 | 21688 | 25 | 4 |
| 22 | P35700 | Prdx1 | Peroxiredoxin-1 | 317 | 2.77 | 22390 | 0.43 | 7 |
| 23 | Q64433 | Hspe1 | 10 kDa heat shock protein, mitochondrial | 268 | 4.22 | 10956 | 0.53 | 5 |
| 24 | Q03265 | Atp5a1 | ATP synthase subunit alpha, mitochondrial | 1583 | 3.54 | 59830 | 44 | 20 |
| 25 | P11835 | Itgb2 | Integrin beta-2 | 2360 | 5.07 | 88031 | 53 | 34 |
| 26 | Q9JKR6 | Hyou1 | Hypoxia up-regulated protein 1 | 3422 | 4.86 | 111340 | 58 | 42 |
| 27 | P11942 | Cd3g | T-cell surface glycoprotein CD3 gamma chain | 346 | 1.97 | 20450 | 32 | 6 |
| 28 | P47911 | Rpl6 | 60S ribosomal protein L6 | 602 | 3.27 | 33546 | 34 | 12 |
| 29 | Q61753 | Phgdh | D-3-phosphoglycerate dehydrogenase | 1314 | 2.97 | 57347 | 36 | 18 |
| 30 | P08113 | Hsp90b1 | Endoplasmin | 1787 | 2.84 | 92703 | 39 | 26 |
| 31 | O08573-2 | Lgals9 | Isoform Short of Galectin-9 | 814 | 3.05 | 40281 | 34 | 9 |
| 32 | P16045 | Lgals1 | Galectin-1 | 448 | 5.93 | 15198 | 53 | 6 |
| 33 | P01851 | Trbc1 | T-cell receptor beta-2 chain C region | 69 | 0.64 | 19129 | 33 | 2 |
| 34 | P10852 | Slc3a2 | 4F2 cell-surface antigen heavy chain | 2047 | 5.54 | 62315 | 44 | 25 |
| 35 | P14148 | Rpl7 | 60S ribosomal protein L7 | 233 | 1.24 | 32564 | 2 | 7 |
| 36 | P61358 | Rpl27 | 60S ribosomal protein L27 | 144 | 1.55 | 15788 | 21 | 3 |
| 37 | Q9JJI8 | Rpl38 | 60S ribosomal protein L38 | 186 | 4.72 | 8256 | 5 | 4 |
| 38 | P99024 | Tubb5 | Tubulin beta-5 chain | 3401 | 17.77 | 50095 | 71 | 4 |
| 39 | P62751 | Rpl23a | 60S ribosomal protein L23a | 195 | 1.85 | 17684 | 27 | 4 |
| 40 | P56480 | Atp5b | ATP synthase subunit beta, mitochondrial | 2570 | 5.97 | 56265 | 6 | 21 |
| 41 | P05064 | Aldoa | Fructose-bisphosphate aldolase A | 1153 | 5.02 | 39787 | 57 | 13 |
| 42 | P60843 | Eif4a1 | Eukaryotic initiation factor 4A-I | 1310 | 4.06 | 46353 | 41 | 14 |
| 43 | P17182 | Eno1 | Alpha-enolase | 1573 | 4.27 | 47453 | 47 | 15 |
| 44 | P06151 | Ldha | L-lactate dehydrogenase A chain | 624 | 2.08 | 40075 | 34 | 10 |
| 45 | P18572 | Bsg | Basigin | 727 | 2.07 | 29941 | 5 | 9 |
| 46 | P84099 | Rpl19 | 60S ribosomal protein L19 | 275 | 0.61 | 23347 | 13 | 2 |
| 47 | Q9CZN7 | Shmt2 | Serine hydroxymethyltransferase | 964 | 3.08 | 56237 | 43 | 16 |
| 48 | Q9D8E6 | Rpl4 | 60S ribosomal protein L4 | 819 | 2.29 | 47409 | 38 | 14 |
| 49 | P61255 | Rpl26 | 60S ribosomal protein L26 | 184 | 1.92 | 17248 | 32 | 5 |
| 50 | P21995 | Emb | Embigin | 804 | 3.95 | 37554 | 38 | 11 |

**Supplementary Table 3. SH-IQ:** **50 most abundant proteins identified after avidin purification.**

2B4 T cells were reduced with TCEP and free cysteines labelled with MPB. The sample was then enriched for biotinylated proteins with avidin beads and prepared for mass spectrometry analysis. The results were searched using the CPF Pipeline (10 ppm precursor tolerance, 0.02 Da fragment tolerance, 2 missed cleavages). SINQ (FDR 1%) analysis was performed to determine relative protein abundances. For the cell surface proteins listed in the table the following information is shown: Abundance rank (1 indicating the most abundant protein in the sample), UniProt accession number, gene name, Mascot score, emPAI (exponentially modified protein abundance index), molecular weight of the protein in Da, sequence coverage and unique peptide sequences identified.

| **Abundance Rank** | **Accession** | **Gene** | **Description** | **Mascot**  **Score** | **emPAI** | **Mass (Da)** | **Sequence coverage (%)** | **Unique peptides** |
| --- | --- | --- | --- | --- | --- | --- | --- | --- |
| 1 | P63017 | Hspa8 | Heat shock cognate 71 kDa protein | 5994 | 24.62 | 70827 | 66 | 33 |
| 2 | P99027 | Rplp2 | 60S acidic ribosomal protein P2 | 1256 | 35.85 | 11644 | 96 | 10 |
| 3 | P06800 | Ptprc | Receptor-type tyrosine-protein phosphatase C | 8292 | 15.83 | 128489 | 63 | 73 |
| 4 | P63038 | Hspd1 | 60 kDa heat shock protein, mitochondrial | 6210 | 62.51 | 60917 | 77 | 49 |
| 5 | P27773 | Pdia3 | Protein disulfide-isomerase A3 | 2948 | 15.41 | 56643 | 67 | 40 |
| 6 | P11499 | Hsp90ab1 | Heat shock protein HSP 90-beta | 5430 | 14.84 | 83229 | 62 | 34 |
| 7 | P10126 | Eef1a1 | Elongation factor 1-alpha 1 | 2970 | 9.52 | 50082 | 77 | 26 |
| 8 | P35700 | Prdx1 | Peroxiredoxin-1 | 836 | 9.9 | 22162 | 68 | 13 |
| 9 | P56480 | Atp5b | ATP synthase subunit beta, mitochondrial | 3205 | 8.73 | 56265 | 66 | 24 |
| 10 | Q6ZWZ6 | Rps12 | 40S ribosomal protein S12 | 441 | 5.03 | 14505 | 58 | 7 |
| 11 | P06151 | Ldha | L-lactate dehydrogenase A chain | 1464 | 7.98 | 39733 | 71 | 18 |
| 12 | P11835 | Itgb2 | Integrin beta-2 | 3013 | 7.62 | 84838 | 65 | 45 |
| 13 | P47962 | Rpl5 | 60S ribosomal protein L5 | 1258 | 5.99 | 34379 | 4 | 13 |
| 14 | Q99MN9 | Pccb | Propionyl-CoA carboxylase beta chain, mitochondrial | 2625 | 6.12 | 58372 | 58 | 25 |
| 15 | E9Q5M7 | Itgal | Integrin alpha-L | 4631 | 4.43 | 128233 | 57 | 51 |
| 16 | Q91ZA3 | Pcca | Propionyl-CoA carboxylase alpha chain, mitochondrial | 3406 | 6.62 | 79871 | 6 | 39 |
| 17 | Q9CPQ1 | Cox6c | Cytochrome c oxidase subunit 6C | 146 | 3.5 | 8464 | 26 | 3 |
| 18 | Q03265 | Atp5a1 | ATP synthase subunit alpha, mitochondrial | 2719 | 6.22 | 59716 | 65 | 31 |
| 19 | P20029 | Hspa5 | 78 kDa glucose-regulated protein | 3411 | 6.8 | 72377 | 5 | 35 |
| 20 | Q8VEM8 | Slc25a3 | Phosphate carrier protein, mitochondrial | 847 | 2.55 | 39711 | 27 | 9 |
| 21 | Q61937 | Npm1 | Nucleophosmin | 1268 | 2.81 | 32540 | 37 | 9 |
| 22 | P17751 | Tpi1 | Triosephosphate isomerase | 1007 | 3.75 | 32171 | 45 | 11 |
| 23 | P18760 | Cfl1 | Cofilin-1 | 379 | 1.91 | 18548 | 54 | 6 |
| 24 | Q61753 | Phgdh | D-3-phosphoglycerate dehydrogenase | 2048 | 5.35 | 56549 | 46 | 24 |
| 25 | Q99LP6 | Grpel1 | GrpE protein homolog 1, mitochondrial | 511 | 5.77 | 24292 | 67 | 13 |
| 26 | P10639 | Txn | Thioredoxin | 317 | 4.28 | 11668 | 49 | 6 |
| 27 | Q6ZWX6 | Eif2s1 | Eukaryotic translation initiation factor 2 subunit 1 | 1341 | 8.28 | 36085 | 7 | 19 |
| 28 | Q8BG32 | Psmd11 | 26S proteasome non-ATPase regulatory subunit 11 | 1309 | 6.29 | 47407 | 6 | 27 |
| 29 | Q9Z0N1 | Eif2s3x | Eukaryotic translation initiation factor 2 subunit 3, X-linked | 1031 | 2.07 | 51033 | 44 | 15 |
| 30 | P17918 | Pcna | Proliferating cell nuclear antigen | 753 | 4.7 | 28766 | 59 | 10 |
| 31 | G3UX26 | Vdac2 | Voltage-dependent anion-selective channel protein 2 (Fragment) | 992 | 5.46 | 30427 | 57 | 13 |
| 32 | Q8R180 | Ero1l | ERO1-like protein alpha | 1312 | 3.75 | 54050 | 5 | 22 |
| 33 | P58252 | Eef2 | Elongation factor 2 | 3078 | 4.91 | 95253 | 59 | 42 |
| 34 | P16045 | Lgals1 | Galectin-1 | 511 | 6.23 | 14856 | 61 | 7 |
| 35 | P08003 | Pdia4 | Protein disulfide-isomerase A4 | 2092 | 6.54 | 71938 | 47 | 33 |
| 36 | P14685 | Psmd3 | 26S proteasome non-ATPase regulatory subunit 3 | 1046 | 2.39 | 60680 | 47 | 21 |
| 37 | P09528 | Fth1 | Ferritin heavy chain | 384 | 3.8 | 21053 | 46 | 10 |
| 38 | P38647 | Hspa9 | Stress-70 protein, mitochondrial | 3491 | 9.47 | 73416 | 63 | 39 |
| 39 | P62908 | Rps3 | 40S ribosomal protein S3 | 607 | 3.48 | 26657 | 46 | 10 |
| 40 | Q99JI4 | Psmd6 | 26S proteasome non-ATPase regulatory subunit 6 | 1463 | 6.35 | 45507 | 59 | 25 |
| 41 | P26516 | Psmd7 | 26S proteasome non-ATPase regulatory subunit 7 | 877 | 2.61 | 36517 | 5 | 12 |
| 42 | Q9WVJ2 | Psmd13 | 26S proteasome non-ATPase regulatory subunit 13 | 1421 | 4.21 | 42782 | 48 | 17 |
| 43 | P63325 | Rps10 | 40S ribosomal protein S10 | 412 | 2.4 | 18904 | 42 | 6 |
| 44 | P47955 | Rplp1 | 60S acidic ribosomal protein P1 | 255 | 2.1 | 11468 | 67 | 3 |
| 45 | P09103 | P4hb | Protein disulfide-isomerase | 1408 | 3.38 | 57023 | 53 | 24 |
| 46 | P67778 | Phb | Prohibitin | 647 | 3.3 | 29802 | 64 | 13 |
| 47 | P60867 | Rps20 | 40S ribosomal protein S20 | 313 | 1.08 | 13364 | 24 | 3 |
| 48 | P51881 | Slc25a5 | ADP/ATP translocase 2 | 828 | 3.6 | 32910 | 47 | 9 |
| 49 | P99024 | Tubb5 | Tubulin beta-5 chain | 3228 | 12.16 | 49639 | 72 | 5 |
| 49 | P99024 | Tubb5 | Tubulin beta-5 chain | 3228 | 12.16 | 49639 | 72 | 5 |
| 50 | S4R1W1 | Gm3839 | Glyceraldehyde-3-phosphate dehydrogenase | 1506 | 12.52 | 38629 | 66 | 18 |

**Supplementary Table 4.SH-IQ:** **50 most abundant proteins identified after tandem lectin-avidin purification.**

2B4 T cells were reduced with TCEP and free cysteines labelled with MPB. The sample was then enriched for biotinylated glycoproteins by tandem lectin-avidin purification and prepared for mass spectrometry analysis. The results were searched using the CPF Pipeline (10 ppm precursor tolerance, 0.02 Da fragment tolerance, 2 missed cleavages). SINQ (FDR 1%) analysis was performed to determine relative protein abundances. For the cell surface proteins listed in the table the following information is shown: Abundance rank (1 indicating the most abundant protein in the sample), UniProt accession number, gene name, Mascot score, emPAI (exponentially modified protein abundance index), molecular weight of the protein in Da, sequence coverage and unique peptide sequences identified.

| **Abundance Rank** | **Accession** | **Gene** | **Description** | **Mascot**  **Score** | **emPAI** | **Mass (Da)** | **Sequence coverage (%)** | **Unique peptides** |
| --- | --- | --- | --- | --- | --- | --- | --- | --- |
| 1 | P06800 | Ptprc | Receptor-type tyrosine-protein phosphatase C | 9863 | 40.46 | 128489 | 55 | 66 |
| 2 | P11835 | Itgb2 | Integrin beta-2 | 5372 | 34.63 | 84838 | 63 | 46 |
| 3 | E9Q5M7 | Itgal | Integrin alpha-L | 6004 | 11.82 | 128233 | 53 | 55 |
| 4 | P15379-10 | Cd44 | Isoform 8 of CD44 antigen | 2887 | 2.72 | 72043 | 16 | 10 |
| 5 | P43406 | Itgav | Integrin alpha-V | 3819 | 16.03 | 115287 | 51 | 53 |
| 6 | P10852 | Slc3a2 | 4F2 cell-surface antigen heavy chain | 2121 | 10.41 | 62201 | 39 | 24 |
| 7 | Q62351 | Tfrc | Transferrin receptor protein 1 | 1779 | 8.12 | 85677 | 43 | 27 |
| 8 | P38647 | Hspa9 | Stress-70 protein, mitochondrial | 3752 | 29.51 | 73416 | 58 | 40 |
| 9 | P99027 | Rplp2 | 60S acidic ribosomal protein P2 | 694 | 78.25 | 11644 | 95 | 9 |
| 10 | O54890 | Itgb3 | Integrin beta-3 | 2419 | 6.97 | 86681 | 47 | 30 |
| 11 | P10126 | Eef1a1 | Elongation factor 1-alpha 1 | 756 | 3.12 | 50082 | 39 | 13 |
| 12 | P35700 | Prdx1 | Peroxiredoxin-1 | 498 | 31.15 | 18858 | 53 | 11 |
| 13 | P56382 | Atp5e | ATP synthase subunit epsilon, mitochondrial | 52 | 1.16 | 5834 | 15 | 1 |
| 14 | P10404 | 1 SV | MLV-related proviral Env polyprotein | 2170 | 8.42 | 69569 | 43 | 16 |
| 15 | P09055 | Itgb1 | Integrin beta-1 | 2265 | 7.58 | 88173 | 45 | 33 |
| 16 | P56480 | Atp5b | ATP synthase subunit beta, mitochondrial | 1984 | 12.51 | 56265 | 47 | 20 |
| 17 | Q03265 | Atp5a1 | ATP synthase subunit alpha, mitochondrial | 1290 | 6.83 | 59716 | 45 | 21 |
| 18 | Q3TB92 | Milr1 | Allergin-1 | 1032 | 30.26 | 27349 | 53 | 11 |
| 19 | O08573-2 | Lgals9 | Isoform Short of Galectin-9 | 708 | 3.66 | 39882 | 3 | 9 |
| 20 | P20029 | Hspa5 | 78 kDa glucose-regulated protein | 1899 | 6.59 | 72377 | 48 | 27 |
| 21 | P48962 | Slc25a4 | ADP/ATP translocase 1 | 282 | 3.19 | 32883 | 33 | 4 |
| 22 | D3YZZ5 | Tmed7 | Protein Tmed7 | 311 | 2.06 | 25185 | 24 | 6 |
| 23 | P04223-2 | H2-K1 | Isoform 2 of H-2 class I histocompatibility antigen, K-K alpha chain | 709 | 4.71 | 40604 | 0.39 | 9 |
| 24 | Q8C129 | Lnpep | Leucyl-cystinyl aminopeptidase | 2543 | 6.55 | 117229 | 0.4 | 37 |
| 25 | P06151 | Ldha | L-lactate dehydrogenase A chain | 526 | 2.69 | 39733 | 28 | 9 |
| 26 | P27773 | Pdia3 | Protein disulfide-isomerase A3 | 1020 | 7.06 | 56643 | 45 | 23 |
| 27 | P63017 | Hspa8 | Heat shock cognate 71 kDa protein | 1688 | 8.07 | 70827 | 52 | 25 |
| 28 | P16045 | Lgals1 | Galectin-1 | 466 | 7.95 | 14856 | 45 | 5 |
| 29 | G3UX26 | Vdac2 | Voltage-dependent anion-selective channel protein 2 (Fragment) | 526 | 3.02 | 30427 | 39 | 8 |
| 30 | Q8VEM8 | Slc25a3 | Phosphate carrier protein, mitochondrial | 337 | 1.58 | 39711 | 22 | 7 |
| 31 | P01831 | Thy1 | Thy-1 membrane glycoprotein | 306 | 5.1 | 18069 | 51 | 6 |
| 32 | P01851 | 1 SV | T-cell receptor beta-2 chain C region | 2170 | 8.42 | 69569 | 43 | 16 |
| 33 | P11499 | Hsp90ab1 | Heat shock protein HSP 90-beta | 2017 | 6.75 | 83229 | 43 | 16 |
| 34 | Q9DB20 | Atp5o | ATP synthase subunit O, mitochondrial | 525 | 8.15 | 23349 | 53 | 8 |
| 35 | P42932 | Cct8 | T-complex protein 1 subunit theta | 1139 | 6.89 | 59518 | 44 | 22 |
| 36 | Q8C145 | Slc39a6 | Zinc transporter ZIP6 | 972 | 1.28 | 86326 | 13 | 12 |
| 37 | Q01965 | Ly9 | T-lymphocyte surface antigen Ly-9 | 1168 | 2.2 | 73096 | 32 | 16 |
| 38 | P14426 | H2-D1 | H-2 class I histocompatibility antigen, D-K alpha chain | 631 | 2.59 | 40594 | 3 | 4 |
| 39 | Q6ZWZ6 | Rps12 | 40S ribosomal protein S12 | 166 | 2.6 | 14505 | 34 | 4 |
| 40 | P97370 | Atp1b3 | Sodium/potassium-transporting ATPase subunit beta-3 | 149 | 0.56 | 31755 | 18 | 3 |
| 41 | P08113 | Hsp90b1 | Endoplasmin | 987 | 1.93 | 92418 | 32 | 18 |
| 42 | P51881 | Slc25a5 | ADP/ATP translocase 2 | 345 | 3.84 | 32910 | 31 | 4 |
| 43 | Q02242 | Pdcd1 | Programmed cell death protein 1 | 651 | 3.38 | 31822 | 32 | 8 |
| 44 | Q61753 | Phgdh | D-3-phosphoglycerate dehydrogenase | 811 | 3.9 | 56549 | 37 | 17 |
| 45 | P24668 | M6pr | Cation-dependent mannose-6-phosphate receptor | 304 | 1.47 | 31152 | 3 | 6 |
| 46 | Q6P5F6 | Slc39a10 | Zinc transporter ZIP10 | 518 | 1.23 | 94335 | 17 | 14 |
| 47 | P11370 | Fv4 | Retrovirus-related Env polyprotein from Fv-4 locus | 806 | 1.01 | 74406 | 17 | 4 |
| 48 | Q62179 | Sema4b | Semaphorin-4B | 819 | 1.68 | 91335 | 25 | 5 |
| 49 | Q61543 | Glg1 | Golgi apparatus protein 1 | 2049 | 4.58 | 132265 | 35 | 36 |
| 50 | Q80V42 | Cpm | Carboxypeptidase M | 773 | 3.07 | 50524 | 35 | 14 |

**Supplementary Table 5.** **Quantitation of labile disulfide bond reduction in leukocyte cell surface proteins after TCEP reduction.**

The listed 163 proteins have been identified in three biological replicates. The average fold change (AFC) has been calculated on a minimum of two unique peptides per protein. Limma analysis was used to analyse the differential protein abundance between control and reduced samples. The P-values were adjusted for multiple testing applying Benjamini-Hochberg correction. Cell surface proteins are indicated in black, intracellular proteins in grey and proteins with known labile disulfide bonds bold. For the cell surface proteins listed in the table the following information is shown: UniProt accession number, gene name, gene description, AFC, log_2_(AFC), log_2_(Average abundance), P-value and adjusted P-value.

| **Accession** | **Gene Name** | **Protein Description** | **Average Fold Change** | **log_2_(AFC)** | **log_2_(Average Abundance)** | **P-value** | **Adjusted P-value** |
| --- | --- | --- | --- | --- | --- | --- | --- |
| Q9CYA0 | Creld2 | Cysteine-rich with EGF-like domain protein 2 | 10.39 | 3.38 | 18.34 | 3.92E-04 | 0.009 |
| Q62469 | Itga2 | Integrin alpha-2 | 9.79 | 3.29 | 22.83 | 3.16E-05 | 0.005 |
| A2APM1 | Cd44 | CD44 antigen | 9.08 | 3.18 | 26.27 | 8.20E-05 | 0.007 |
| Q9QZF2 | Gpc1 | Glypican-1 | 8.86 | 3.15 | 18.33 | 2.23E-04 | 0.008 |
| Q80V42 | Cpm | Carboxypeptidase M | 8.52 | 3.09 | 20.26 | 4.95E-04 | 0.009 |
| P35456 | Plaur | Urokinase plasminogen activator surface receptor | 8.32 | 3.06 | 18.96 | 8.92E-04 | 0.013 |
| O54890 | Itgb3 | Integrin beta-3 | 8.11 | 3.02 | 24.92 | 7.84E-04 | 0.013 |
| Q60846 | Tnfrsf8 | Tumor necrosis factor receptor superfamily member 8, CD30 | 7.80 | 2.96 | 24.22 | 5.10E-04 | 0.009 |
| P09055 | Itgb1 | Integrin beta-1 | 7.31 | 2.87 | 25.39 | 1.79E-04 | 0.008 |
| Q6P5F6 | Slc39a10 | Zinc transporter ZIP10 | 6.02 | 2.59 | 23.87 | 4.21E-04 | 0.009 |
| Q8R2Q8 | Bst2 | Bone marrow stromal antigen 2 | 4.55 | 2.19 | 20.06 | 1.19E-03 | 0.013 |
| Q64151 | Sema4c | Semaphorin-4C | 4.02 | 2.01 | 18.94 | 7.61E-03 | 0.047 |
| Q542I8 | Itgb2 | Integrin beta | 3.86 | 1.95 | 28.20 | 1.03E-03 | 0.013 |
| Q64697 | Ptprcap | Protein tyrosine phosphatase receptor type C-associated protein | 3.77 | 1.92 | 20.23 | 9.53E-03 | 0.049 |
| E9Q5M7 | Itgal | Integrin alpha-L | 3.44 | 1.78 | 28.53 | 9.61E-04 | 0.013 |
| O89001 | Cpd | Carboxypeptidase D | 3.44 | 1.78 | 20.28 | 1.22E-03 | 0.013 |
| O35598 | Adam10 | Disintegrin and metalloproteinase domain-containing protein 10 | 3.24 | 1.70 | 21.76 | 2.75E-03 | 0.028 |
| P01831 | Thy1 | Thy-1 membrane glycoprotein | 3.02 | 1.60 | 22.73 | 6.01E-03 | 0.047 |
| Q3TB92 | Milr1 | Allergin-1 | 2.64 | 1.40 | 23.52 | 7.67E-03 | 0.047 |
| O09126 | Sema4d | Semaphorin-4D | 2.20 | 1.14 | 24.23 | 7.58E-03 | 0.047 |
| A2AN91 | Susd1 | Protein Susd1 | 1.94 | 0.96 | 21.26 | 4.86E-03 | 0.041 |
| Q91VK1 | Bzw2 | Basic leucine zipper and W2 domain-containing protein 2 | 1.71 | 0.78 | 18.22 | 2.53E-01 | 0.389 |
| P16045 | Lgals1 | Galectin-1 | 1.68 | 0.75 | 19.65 | 4.21E-02 | 0.114 |
| Q8K094 | Pvr | Poliovirus receptor | 1.62 | 0.69 | 21.79 | 1.17E-02 | 0.058 |
| Q61503 | Nt5e | 5'-nucleotidase | 1.59 | 0.66 | 19.76 | 2.28E-01 | 0.361 |
| Q02242 | Pdcd1 | Programmed cell death protein 1 | 1.54 | 0.62 | 22.22 | 2.59E-02 | 0.085 |
| Q8BMS1 | Hadha | Trifunctional enzyme subunit alpha, mitochondrial | 1.53 | 0.61 | 21.80 | 1.75E-02 | 0.075 |
| Q3U0X8 | Cd96 | T-cell surface protein tactile | 1.49 | 0.58 | 18.75 | 1.03E-01 | 0.207 |
| P24668 | M6pr | Cation-dependent mannose-6-phosphate receptor | 1.49 | 0.57 | 21.90 | 7.16E-02 | 0.169 |
| P04202 | Tgfb1 | Transforming growth factor beta-1 | 1.34 | 0.42 | 20.50 | 1.01E-01 | 0.205 |
| P51881 | Slc25a5 | ADP/ATP translocase 2 | 1.33 | 0.41 | 21.58 | 3.25E-01 | 0.452 |
| Q07797 | Lgals3bp | Galectin-3-binding protein | 1.32 | 0.40 | 20.93 | 2.86E-01 | 0.413 |
| P48962 | Slc25a4 | ADP/ATP translocase 1 | 1.32 | 0.40 | 19.12 | 1.76E-01 | 0.302 |
| P35330 | Icam2 | Intercellular adhesion molecule 2 | 1.31 | 0.39 | 22.06 | 1.93E-01 | 0.325 |
| P01735 | 4 SV | T-cell receptor beta chain V region 86T1 | 1.23 | 0.30 | 20.73 | 3.06E-01 | 0.433 |
| Q60943 | Il17ra | Interleukin-17 receptor A | 1.21 | 0.28 | 17.73 | 3.31E-01 | 0.453 |
| P28843 | Dpp4 | Dipeptidyl peptidase 4 | 1.21 | 0.27 | 24.88 | 5.03E-01 | 0.622 |
| Q9QUM4-2 | Slamf1 | Isoform Short of Signaling lymphocytic activation molecule | 1.21 | 0.27 | 19.98 | 6.07E-01 | 0.717 |
| Q8K4F0 | Cd226 | CD226 antigen | 1.19 | 0.25 | 19.59 | 2.66E-01 | 0.398 |
| Q9JJF9 | Sppl2a | Signal peptide peptidase-like 2A | 1.17 | 0.23 | 18.13 | 5.19E-01 | 0.636 |
| P04235 | Cd3d | T-cell surface glycoprotein CD3 delta chain | 1.17 | 0.22 | 20.59 | 4.76E-01 | 0.597 |
| P35951 | Ldlr | Low-density lipoprotein receptor | 1.16 | 0.22 | 23.97 | 3.59E-01 | 0.480 |
| P35564 | Canx | Calnexin | 1.15 | 0.20 | 20.19 | 4.89E-01 | 0.608 |
| P10852-2 | Slc3a2 | Isoform 2 of 4F2 cell-surface antigen heavy chain | 1.15 | 0.20 | 27.47 | 3.25E-01 | 0.452 |
| P19001 | Krt19 | Keratin, type I cytoskeletal 19 | 1.11 | 0.16 | 18.87 | 8.49E-01 | 0.898 |
| Q91WD0-2 | Gpr108 | Isoform 2 of Protein GPR108 | 1.09 | 0.13 | 19.89 | 7.45E-01 | 0.838 |
| Q64735-2 | Cr1l | Isoform 2 of Complement component receptor 1-like protein | 1.09 | 0.12 | 23.07 | 5.60E-01 | 0.676 |
| Q8R5L1 | C1qbp | Complement component 1 Q subcomponent-binding protein, mitochondrial | 1.09 | 0.12 | 20.07 | 8.03E-01 | 0.865 |
| Q80VP8 | Tmem106c | Transmembrane protein 106C | 1.08 | 0.11 | 20.00 | 8.07E-01 | 0.865 |
| Q8VD58 | Evi2b | Protein EVI2B | 1.08 | 0.11 | 23.09 | 6.49E-01 | 0.756 |
| Q9EQH2 | Erap1 | Endoplasmic reticulum aminopeptidase 1 | 1.07 | 0.10 | 21.11 | 6.19E-01 | 0.726 |
| Q99JY0 | Hadhb | Trifunctional enzyme subunit beta, mitochondrial | 1.07 | 0.09 | 21.70 | 6.86E-01 | 0.788 |
| B1AQR8 | Lgals9 | Galectin | 1.05 | 0.07 | 25.83 | 7.70E-01 | 0.848 |
| P23242 | Gja1 | Gap junction alpha-1 protein | 1.03 | 0.04 | 20.48 | 8.40E-01 | 0.895 |
| Q9CYN9 | Atp6ap2 | Renin receptor | 1.03 | 0.04 | 19.20 | 8.59E-01 | 0.904 |
| P07356 | Anxa2 | Annexin A2 | 1.02 | 0.03 | 19.75 | 8.97E-01 | 0.925 |
| P70289 | Ptprv | Receptor-type tyrosine-protein phosphatase V | 1.02 | 0.03 | 20.40 | 8.93E-01 | 0.925 |
| P51863 | Atp6v0d1 | V-type proton ATPase subunit d 1 | 1.01 | 0.01 | 21.30 | 9.53E-01 | 0.977 |
| P24161 | Cd247 | T-cell surface glycoprotein CD3 zeta chain | 1.01 | 0.01 | 20.71 | 9.90E-01 | 0.997 |
| P22646 | Cd3e | T-cell surface glycoprotein CD3 epsilon chain | 1.01 | 0.01 | 22.92 | 9.80E-01 | 0.996 |
| S4R1M0 | Ptprc | Receptor-type tyrosine-protein phosphatase C | 1.00 | 0.00 | 30.79 | 1.00E+00 | 1.000 |
| Q8VE99 | Ccdc115 | Coiled-coil domain-containing protein 115 | 1.00 | 0.00 | 19.18 | 9.84E-01 | 0.996 |
| P67778 | Phb | Prohibitin | 0.98 | -0.03 | 18.03 | 8.67E-01 | 0.905 |
| P28658 | Atxn10 | Ataxin-10 | 0.96 | -0.05 | 20.71 | 7.63E-01 | 0.846 |
| Q8K201 | Kct2 | Keratinocyte-associated transmembrane protein 2 | 0.96 | -0.06 | 19.68 | 7.80E-01 | 0.853 |
| Q9ET30 | Tm9sf3 | Transmembrane 9 superfamily member 3 | 0.96 | -0.06 | 19.30 | 7.58E-01 | 0.846 |
| O35114 | Scarb2 | Lysosome membrane protein 2 | 0.95 | -0.07 | 21.40 | 7.44E-01 | 0.838 |
| Q9EPS3 | Glce | D-glucuronyl C5-epimerase | 0.93 | -0.11 | 19.18 | 7.94E-01 | 0.862 |
| P01851 | 1 SV | T-cell receptor beta-2 chain C region | 0.91 | -0.14 | 21.43 | 5.34E-01 | 0.650 |
| F8VQJ3 | Lamc1 | Laminin subunit gamma-1 | 0.91 | -0.14 | 21.61 | 6.01E-01 | 0.715 |
| Q68FM6 | Elfn2 | Protein phosphatase 1 regulatory subunit 29 | 0.90 | -0.16 | 21.43 | 4.12E-01 | 0.530 |
| Q9D771 | Tmem206 | Transmembrane protein 206 | 0.90 | -0.16 | 21.12 | 7.08E-01 | 0.807 |
| Q91VD9 | Ndufs1 | NADH-ubiquinone oxidoreductase 75 kDa subunit, mitochondrial | 0.90 | -0.16 | 18.10 | 4.25E-01 | 0.541 |
| A2A7A7 | H6pd | GDH/6PGL endoplasmic bifunctional protein | 0.90 | -0.16 | 19.64 | 6.79E-01 | 0.785 |
| Q99KF1 | Tmed9 | Transmembrane emp24 domain-containing protein 9 | 0.87 | -0.21 | 21.51 | 2.77E-01 | 0.406 |
| Z4YK56 | AU040320 | Protein AU040320 | 0.86 | -0.22 | 18.91 | 4.13E-01 | 0.530 |
| P01734 | 4 SV | T-cell receptor beta chain V region 3H.25 | 0.85 | -0.23 | 19.89 | 4.39E-01 | 0.555 |
| Q8VDL4-3 | Adpgk | Isoform 3 of ADP-dependent glucokinase | 0.84 | -0.25 | 20.12 | 2.62E-01 | 0.395 |
| Q80XN0 | Bdh1 | D-beta-hydroxybutyrate dehydrogenase, mitochondrial | 0.83 | -0.27 | 19.25 | 2.56E-01 | 0.390 |
| P17439 | Gba | Glucosylceramidase | 0.82 | -0.29 | 20.17 | 3.92E-01 | 0.511 |
| P17918 | Pcna | Proliferating cell nuclear antigen | 0.82 | -0.29 | 19.78 | 2.14E-01 | 0.346 |
| Q9QYJ0 | Dnaja2 | DnaJ homolog subfamily A member 2 | 0.81 | -0.31 | 20.10 | 3.62E-01 | 0.480 |
| Q3TAA8 | Pigu | Phosphatidylinositol glycan anchor biosynthesis class U protein | 0.79 | -0.34 | 19.74 | 1.23E-01 | 0.237 |
| Q920A5 | Scpep1 | Retinoid-inducible serine carboxypeptidase | 0.79 | -0.34 | 19.89 | 9.82E-02 | 0.203 |
| Q3U0P5 | Entpd6 | Ectonucleoside triphosphate diphosphohydrolase 6, isoform CRA_a | 0.78 | -0.35 | 21.61 | 2.40E-01 | 0.376 |
| Q7JCZ1 | mt-Co2 | Cytochrome c oxidase subunit 2 | 0.78 | -0.36 | 18.50 | 2.14E-01 | 0.346 |
| Q8HWA3 | Ulbp1 | Protein Ulbp1 | 0.78 | -0.36 | 23.02 | 1.98E-01 | 0.327 |
| Q3TDQ1 | Stt3b | Dolichyl-diphosphooligosaccharide--protein glycosyltransferase subunit STT3B | 0.78 | -0.37 | 20.99 | 3.46E-01 | 0.466 |
| Q3UHN9 | Ndst1 | Bifunctional heparan sulfate N-deacetylase/N-sulfotransferase 1 | 0.77 | -0.37 | 16.92 | 5.68E-01 | 0.680 |
| G5E902 | Slc25a3 | MCG10343, isoform CRA_b | 0.77 | -0.37 | 22.89 | 1.24E-01 | 0.237 |
| O89051 | Itm2b | Integral membrane protein 2B | 0.77 | -0.37 | 21.32 | 1.76E-01 | 0.302 |
| Q3U9G9 | Lbr | Lamin-B receptor | 0.77 | -0.38 | 19.85 | 2.79E-01 | 0.406 |
| Q8C7U7 | Galnt6 | Polypeptide N-acetylgalactosaminyltransferase 6 | 0.76 | -0.39 | 21.25 | 1.14E-01 | 0.224 |
| Q8BVE3 | Atp6v1h | V-type proton ATPase subunit H | 0.76 | -0.40 | 19.55 | 1.07E-01 | 0.214 |
| E9Q557 | Dsp | Desmoplakin | 0.76 | -0.40 | 21.53 | 3.70E-01 | 0.486 |
| Q60767 | Ly75 | Lymphocyte antigen 75 | 0.74 | -0.43 | 25.22 | 6.97E-02 | 0.167 |
| Q8BMT4-2 | Nrros | Isoform 2 of Negative regulator of reactive oxygen species | 0.74 | -0.43 | 18.75 | 8.87E-02 | 0.190 |
| Q9CRT8 | Xpot | Exportin-T | 0.74 | -0.44 | 19.14 | 7.43E-02 | 0.173 |
| Q792Z1 | Try10 | MCG140784 | 0.74 | -0.44 | 24.34 | 3.27E-01 | 0.452 |
| Q91YQ5 | Rpn1 | Dolichyl-diphosphooligosaccharide--protein glycosyltransferase subunit 1 | 0.74 | -0.44 | 23.95 | 8.08E-02 | 0.181 |
| Q61074 | Ppm1g | Protein phosphatase 1G | 0.73 | -0.45 | 18.75 | 1.40E-01 | 0.251 |
| Q3UXS0 | Scamp3 | Secretory carrier-associated membrane protein 3 | 0.72 | -0.46 | 19.42 | 1.27E-01 | 0.238 |
| O88325 | Naglu | Alpha-N-acetylglucosaminidase | 0.72 | -0.47 | 21.02 | 4.10E-02 | 0.114 |
| Q9CPQ3 | Tomm22 | Mitochondrial import receptor subunit TOM22 homolog | 0.72 | -0.47 | 20.17 | 7.92E-02 | 0.181 |
| Q9JJZ2 | Tuba8 | Tubulin alpha-8 chain | 0.72 | -0.48 | 18.06 | 8.97E-02 | 0.190 |
| P53986 | Slc16a1 | Monocarboxylate transporter 1 | 0.72 | -0.48 | 20.67 | 6.64E-02 | 0.163 |
| P68368 | Tuba4a | Tubulin alpha-4A chain | 0.71 | -0.50 | 20.94 | 2.26E-01 | 0.361 |
| Q60932-2 | Vdac1 | Isoform Mt-VDAC1 of Voltage-dependent anion-selective channel protein 1 | 0.71 | -0.50 | 17.87 | 1.57E-01 | 0.277 |
| Q9WU60 | Atrn | Attractin | 0.70 | -0.51 | 21.15 | 4.81E-02 | 0.129 |
| O35604 | Npc1 | Niemann-Pick C1 protein | 0.70 | -0.52 | 22.95 | 3.80E-02 | 0.109 |
| D3YTP0 | Steap3 | Metalloreductase STEAP3 (Fragment) | 0.69 | -0.54 | 18.83 | 1.39E-01 | 0.251 |
| Q8VBZ3 | Clptm1 | Cleft lip and palate transmembrane protein 1 homolog | 0.69 | -0.54 | 22.67 | 6.13E-02 | 0.154 |
| Q3U1J4 | Ddb1 | DNA damage-binding protein 1 | 0.68 | -0.55 | 19.47 | 4.21E-02 | 0.114 |
| Q8BYI8-2 | Kiaa1467 | Isoform 2 of Uncharacterized protein KIAA1467 | 0.68 | -0.56 | 19.86 | 1.40E-01 | 0.251 |
| P97350 | Pkp1 | Plakophilin-1 | 0.67 | -0.57 | 17.97 | 3.33E-01 | 0.453 |
| Q91UZ6 | D17H6S56E-5 | DNA segment, Chr 17, human D6S56E 5, isoform CRA_c | 0.67 | -0.58 | 22.54 | 3.80E-02 | 0.109 |
| Q8BL63 | Pigk | GPI-anchor transamidase | 0.67 | -0.58 | 20.34 | 2.62E-02 | 0.085 |
| P27046 | Man2a1 | Alpha-mannosidase 2 | 0.66 | -0.60 | 22.02 | 3.27E-02 | 0.100 |
| P70168 | Kpnb1 | Importin subunit beta-1 | 0.66 | -0.60 | 22.83 | 2.22E-02 | 0.079 |
| Q3V3R1 | Mthfd1l | Monofunctional C1-tetrahydrofolate synthase, mitochondrial | 0.66 | -0.61 | 18.66 | 5.85E-02 | 0.151 |
| Q8K2C7-2 | Os9 | Isoform 2 of Protein -9 | 0.66 | -0.61 | 17.04 | 3.82E-02 | 0.109 |
| P99027 | Rplp2 | 60S acidic ribosomal protein P2 | 0.65 | -0.62 | 22.82 | 1.82E-02 | 0.076 |
| Q91XA2 | Golm1 | Golgi membrane protein 1 | 0.65 | -0.62 | 22.84 | 8.11E-02 | 0.181 |
| Q9D8L3 | Ssr4 | Signal sequence receptor, delta | 0.65 | -0.63 | 22.03 | 1.98E-02 | 0.079 |
| Q9CY50 | Ssr1 | Translocon-associated protein subunit alpha | 0.65 | -0.63 | 23.68 | 2.13E-02 | 0.079 |
| Q9QY81 | Nup210 | Nuclear pore membrane glycoprotein 210 | 0.65 | -0.63 | 26.90 | 8.24E-02 | 0.182 |
| Q9DBH5 | Lman2 | Vesicular integral-membrane protein VIP36 | 0.64 | -0.64 | 18.86 | 2.91E-01 | 0.415 |
| Q99KV1 | Dnajb11 | DnaJ homolog subfamily B member 11 | 0.64 | -0.65 | 19.25 | 6.69E-02 | 0.163 |
| P01849 | Tcra | T-cell receptor alpha chain C region | 0.63 | -0.66 | 23.56 | 3.66E-02 | 0.109 |
| P62814 | Atp6v1b2 | V-type proton ATPase subunit B, brain isoform | 0.63 | -0.67 | 20.05 | 2.45E-02 | 0.085 |
| Q9D1D4 | Tmed10 | Transmembrane emp24 domain-containing protein 10 | 0.62 | -0.68 | 23.03 | 2.83E-02 | 0.091 |
| Q5XJY5 | Arcn1 | Coatomer subunit delta | 0.62 | -0.68 | 19.54 | 1.80E-01 | 0.306 |
| Q99LL3 | Chst12 | Carbohydrate sulfotransferase 12 | 0.61 | -0.72 | 20.91 | 2.24E-02 | 0.079 |
| Q9D1K2 | Atp6v1f | V-type proton ATPase subunit F | 0.60 | -0.74 | 19.40 | 1.29E-02 | 0.060 |
| O54734 | Ddost | Dolichyl-diphosphooligosaccharide--protein glycosyltransferase 48 kDa subunit | 0.59 | -0.75 | 23.75 | 1.47E-02 | 0.065 |
| O09159 | Man2b1 | Lysosomal alpha-mannosidase | 0.59 | -0.77 | 19.73 | 6.10E-02 | 0.154 |
| Q62351 | Tfrc | Transferrin receptor protein 1 | 0.59 | -0.77 | 27.45 | 1.74E-01 | 0.302 |
| P11942 | Cd3g | T-cell surface glycoprotein CD3 gamma chain | 0.58 | -0.79 | 21.78 | 1.25E-01 | 0.237 |
| O55029 | Copb2 | Coatomer subunit beta' | 0.58 | -0.80 | 18.94 | 1.99E-01 | 0.327 |
| G3UYZ1 | Igsf8 | Immunoglobulin superfamily member 8 | 0.56 | -0.83 | 19.56 | 9.85E-02 | 0.203 |
| Q61753 | Phgdh | D-3-phosphoglycerate dehydrogenase | 0.56 | -0.83 | 23.44 | 8.68E-03 | 0.047 |
| E9Q0F0 | Krt78 | Protein Krt78 | 0.56 | -0.84 | 24.53 | 1.37E-01 | 0.251 |
| P97370 | Atp1b3 | Sodium/potassium-transporting ATPase subunit beta-3 | 0.56 | -0.84 | 24.93 | 8.00E-03 | 0.047 |
| Q03265 | Atp5a1 | ATP synthase subunit alpha, mitochondrial | 0.56 | -0.85 | 24.60 | 6.68E-03 | 0.047 |
| D3YU17 | Ncln | Nicalin | 0.55 | -0.86 | 20.32 | 8.84E-03 | 0.047 |
| P09405 | Ncl | Nucleolin | 0.55 | -0.86 | 20.39 | 3.19E-02 | 0.100 |
| Q8K297 | Colgalt1 | Procollagen galactosyltransferase 1 | 0.55 | -0.87 | 21.53 | 9.02E-03 | 0.047 |
| Q9Z2K1 | Krt16 | Keratin, type I cytoskeletal 16 | 0.54 | -0.88 | 21.54 | 4.95E-02 | 0.130 |
| Q3TTY5 | Krt2 | Keratin, type II cytoskeletal 2 epidermal | 0.53 | -0.91 | 23.62 | 2.49E-01 | 0.386 |
| Q921V5 | Mgat2 | Alpha-1,6-mannosyl-glycoprotein 2-beta-N-acetylglucosaminyltransferase | 0.53 | -0.91 | 19.43 | 2.23E-02 | 0.079 |
| P17809 | Slc2a1 | Solute carrier family 2, facilitated glucose transporter member 1 | 0.52 | -0.93 | 18.99 | 2.73E-01 | 0.405 |
| Q9JKR6 | Hyou1 | Hypoxia up-regulated protein 1 | 0.52 | -0.95 | 23.81 | 4.70E-03 | 0.041 |
| P56480 | Atp5b | ATP synthase subunit beta, mitochondrial | 0.51 | -0.98 | 25.99 | 9.00E-03 | 0.047 |
| Q64518-2 | Atp2a3 | Isoform SERCA3A of Sarcoplasmic/endoplasmic reticulum calcium ATPase 3 | 0.50 | -0.99 | 19.06 | 2.50E-02 | 0.085 |
| Q01965 | Ly9 | T-lymphocyte surface antigen Ly-9 | 0.50 | -1.00 | 25.36 | 5.01E-03 | 0.041 |
| Q8VCS3 | Fam20b | Glycosaminoglycan xylosylkinase | 0.48 | -1.05 | 19.99 | 1.47E-02 | 0.065 |
| P04104 | Krt1 | Keratin, type II cytoskeletal 1 | 0.47 | -1.08 | 24.36 | 8.76E-02 | 0.190 |
| P20029 | Hspa5 | 78 kDa glucose-regulated protein | 0.47 | -1.08 | 26.37 | 1.26E-02 | 0.060 |
| Q9JLF6 | Tgm1 | Protein-glutamine gamma-glutamyltransferase K | 0.47 | -1.09 | 17.70 | 7.75E-03 | 0.047 |
| Q6GQT9 | Nomo1 | Nodal modulator 1 | 0.45 | -1.16 | 25.00 | 2.14E-02 | 0.079 |
| P07724 | Alb | Serum albumin | 0.37 | -1.45 | 22.83 | 2.06E-02 | 0.079 |
| P38647 | Hspa9 | Stress-70 protein, mitochondrial | 0.26 | -1.96 | 28.28 | 2.48E-04 | 0.008 |
| Q9CPN9 | 2210010C04Rik | Protein 2210010C04Rik | 0.15 | -2.71 | 27.18 | 2.91E-03 | 0.028 |

**Supplementary Table 6.** **Kinetics of labile disulfide bond reduction during immune activation.**

Labile disulfide bond reduction during immune activation was quantified in a MLR using SH-IQ as described before. The data represents the log2(AFC) of two MLRs with an SEM that is smaller than two-fold relative to 4 hours.

| **Acession** | **Description** | **Gene** | **16 h** | | **24 h** | | **48 h** | | **96 h** | |
| --- | --- | --- | --- | --- | --- | --- | --- | --- | --- | --- |
|  |  |  | **AFC** | **SEM** | **AFC** | **SEM** | **AFC** | **SEM** | **AFC** | **SEM** |
| O14672 | Disintegrin and metalloproteinase domain-containing protein 10 | ADAM10 | 0.22 | 0.18 | 1.35 | 0.67 | 1.21 | 0.32 | 1.34 | 0.01 |
| Q99467 | CD180 antigen | CD180 | 1.86 | 0.05 | 0.63 | 0.42 | 0.48 | 0.82 | 0.43 | 0.69 |
| P20273 | B-cell receptor CD22 | CD22 | 1.23 | 0.17 | 1.21 | 0.05 | 1.00 | 0.03 | 0.48 | 0.08 |
| Q9NZQ7 | Programmed cell death 1 ligand 1 | CD274 | 0.39 | 0.05 | -0.16 | 0.21 | 0.17 | 0.10 | -0.43 | 0.16 |
| P28907 | ADP-ribosyl cyclase/cyclic ADP-ribose hydrolase 1 | CD38 | 0.73 | 0.58 | 0.64 | 0.42 | 1.02 | 0.02 | 1.19 | 0.01 |
| P04234 | T-cell surface glycoprotein CD3 delta chain | CD3D | 1.21 | 0.49 | 1.62 | 0.29 | 1.66 | 0.68 | 3.28 | 0.95 |
| H0Y2P0 | CD44 antigen | CD44 | 1.70 | 0.02 | 1.72 | 0.04 | 2.23 | 0.40 | 1.97 | 0.38 |
| P09326 | CD48 antigen | CD48 | 1.75 | 0.24 | 1.57 | 0.15 | 1.41 | 0.03 | 0.99 | 0.42 |
| P06127 | T-cell surface glycoprotein CD5 | CD5 | 1.03 | 0.27 | 1.13 | 0.19 | 1.00 | 0.09 | 0.87 | 0.06 |
| P04233 | HLA class II histocompatibility antigen gamma chain | CD74 | 1.06 | 0.05 | 1.08 | 0.07 | 1.03 | 0.05 | 0.99 | 0.05 |
| P40259 | B-cell antigen receptor complex-associated protein beta chain | CD79B | 1.31 | 0.07 | 2.08 | 0.73 | 0.82 | 0.36 | 0.15 | 0.14 |
| E9PEJ1 | T-cell surface protein tactile | CD96 | 0.63 | 0.58 | 0.11 | 0.21 | -0.01 | 0.16 | 0.04 | 0.08 |
| P48960 | Isoform 3 of CD97 antigen | CD97 | 1.08 | 0.05 | 1.10 | 0.02 | 1.11 | 0.02 | 1.00 | 0.02 |
| P04440 | HLA class II histocompatibility antigen, DP beta 1 chain | HLA-DPB1 | 1.62 | 0.42 | 1.66 | 0.20 | 0.98 | 0.62 | 1.91 | 0.16 |
| P01903 | HLA class II histocompatibility antigen, DR alpha chain | HLA-DRA | 1.16 | 0.12 | 1.13 | 0.03 | 1.24 | 0.05 | 1.20 | 0.03 |
| P13760 | HLA class II histocompatibility antigen, DRB1-4 beta chain | HLA-DRB1 | 1.59 | 0.38 | 1.74 | 0.30 | 2.32 | 0.52 | 1.55 | 0.30 |
| P13762 | HLA class II histocompatibility antigen, DR beta 4 chain | HLA-DRB4 | 1.33 | 0.35 | 1.21 | 0.42 | 1.54 | 0.24 | 1.43 | 0.48 |
| P14625 | Endoplasmin | HSP90B1 | 0.99 | 0.03 | 0.96 | 0.04 | 0.90 | 0.01 | 0.81 | 0.02 |
| P11021 | 78 kDa glucose-regulated protein | HSPA5 | 1.04 | 0.03 | 1.21 | 0.04 | 1.52 | 0.04 | 1.50 | 0.15 |
| P11142 | Heat shock cognate 71 kDa protein | HSPA8 | 1.46 | 0.23 | 1.91 | 0.62 | 1.29 | 0.03 | 1.64 | 0.14 |
| P05362 | Intercellular adhesion molecule 1 | ICAM1 | 1.82 | 0.10 | 1.88 | 0.21 | 2.17 | 0.20 | 2.37 | 0.38 |
| P11717 | Cation-independent mannose-6-phosphate receptor | IGF2R | 1.10 | 0.12 | 1.02 | 0.10 | 1.14 | 0.04 | 1.23 | 0.18 |
| P31785 | Cytokine receptor common subunit gamma | IL2RG | -0.36 | 0.35 | 0.00 | 0.14 | 0.11 | 0.27 | -0.87 | 0.04 |
| P13612 | Integrin alpha-4 | ITGA4 | 1.34 | 0.50 | 1.31 | 0.16 | 2.88 | 0.60 | 1.09 | 0.03 |
| P08648 | Integrin alpha-5 | ITGA5 | 0.95 | 0.03 | 0.40 | 0.19 | 0.57 | 0.25 | 0.01 | 0.22 |
| P20701 | Isoform 2 of Integrin alpha-L | ITGAL | 1.08 | 0.04 | 1.06 | 0.04 | 0.98 | 0.04 | 0.93 | 0.07 |
| P11215 | Isoform 2 of Integrin alpha-M | ITGAM | 1.00 | 0.06 | 0.87 | 0.03 | 0.92 | 0.01 | 0.77 | 0.01 |
| P20702 | Integrin alpha-X | ITGAX | 1.55 | 0.18 | 1.28 | 0.22 | 1.44 | 0.39 | 1.26 | 0.25 |
| P05556 | Integrin beta-1 | ITGB1 | 1.16 | 0.09 | 1.19 | 0.11 | 0.99 | 0.02 | 0.70 | 0.10 |
| P05107 | Integrin beta-2 | ITGB2 | 0.96 | 0.03 | 0.97 | 0.03 | 1.08 | 0.02 | 1.00 | 0.02 |
| P05106 | Integrin beta-3 | ITGB3 | 0.98 | 0.09 | 0.70 | 0.11 | 0.58 | 0.05 | 0.20 | 0.13 |
| P11279 | Lysosome-associated membrane glycoprotein 1 | LAMP1 | 1.46 | 0.29 | 1.07 | 0.09 | 1.42 | 0.22 | 0.72 | 0.04 |
| P13473 | Lysosome-associated membrane glycoprotein 2 | LAMP2 | 0.34 | 0.37 | 1.40 | 0.48 | 1.37 | 0.28 | 0.10 | 0.25 |
| E7ETP9 | Lysosome-associated membrane glycoprotein 3 | LAMP3 | 1.17 | 0.02 | 1.01 | 0.08 | 0.62 | 0.33 | 0.80 | 0.16 |
| P09382 | Galectin-1 | LGALS1 | 0.95 | 0.25 | 1.00 | 0.03 | 0.86 | 0.13 | 0.41 | 0.23 |
| O60449 | Lymphocyte antigen 75 | LY75 | 1.09 | 0.16 | 1.27 | 0.28 | 1.34 | 0.17 | 1.54 | 0.08 |
| P20645 | Cation-dependent mannose-6-phosphate receptor | M6PR | 0.51 | 0.40 | 1.53 | 0.65 | 1.60 | 0.87 | 0.38 | 0.49 |
| P07237 | Protein disulfide-isomerase | P4HB | 0.85 | 0.12 | 0.87 | 0.09 | 0.75 | 0.14 | 1.11 | 0.04 |
| P30101 | Protein disulfide-isomerase A3 | PDIA3 | 1.34 | 0.07 | 0.99 | 0.12 | 1.20 | 0.04 | 0.60 | 0.70 |
| A0A075B738 | Platelet endothelial cell adhesion molecule | PECAM1 | 0.98 | 0.25 | 0.90 | 0.46 | 0.41 | 0.18 | 1.08 | 0.28 |
| Q06830 | Peroxiredoxin-1 | PRDX1 | 0.80 | 0.13 | 1.04 | 0.08 | 0.82 | 0.33 | 1.03 | 0.08 |
| Q12913 | Receptor-type tyrosine-protein phosphatase eta | PTPRJ | 1.04 | 0.06 | 0.97 | 0.03 | 1.06 | 0.13 | 0.96 | 0.08 |
| Q92854 | Semaphorin-4D | SEMA4D | 1.91 | 0.71 | 2.31 | 0.52 | 2.66 | 0.10 | 3.26 | 0.36 |
| J3KPF3 | 4F2 cell-surface antigen heavy chain | SLC3A2 | 1.52 | 0.16 | 1.94 | 0.46 | 2.08 | 0.72 | 2.42 | 0.75 |
| P02730 | Band 3 anion transport protein | SLC4A1 | 0.99 | 0.11 | 1.06 | 0.03 | 0.94 | 0.07 | 0.49 | 0.11 |
| Q01650 | Large neutral amino acids transporter small subunit 1 | SLC7A5 | -0.30 | 0.81 | 0.17 | 0.43 | -0.70 | 0.87 | -1.46 | 0.51 |
| Q99523 | Sortilin | SORT1 | 0.89 | 0.11 | 0.82 | 0.21 | 0.50 | 0.12 | 1.00 | 0.04 |
| Q13488 | V-type proton ATPase 116 kDa subunit a isoform 3 | TCIRG1 | 1.53 | 0.21 | 2.10 | 0.71 | 0.06 | 0.46 | 1.13 | 0.10 |
| P02786 | Transferrin receptor protein 1 | TFRC | 0.91 | 0.54 | 1.58 | 0.01 | 2.60 | 0.37 | 3.54 | 0.76 |
| P01137 | Transforming growth factor beta-1 | TGFB1 | 2.44 | 0.60 | 2.63 | 0.68 | 1.68 | 0.16 | 1.75 | 0.33 |
| P45880 | Voltage-dependent anion-selective channel protein 2 | VDAC2 | 0.73 | 0.22 | 1.23 | 0.29 | 0.59 | 0.43 | 1.22 | 0.49 |
| Q9Y277 | Voltage-dependent anion-selective channel protein 3 | VDAC3 | 0.62 | 0.14 | 0.32 | 0.17 | 0.86 | 0.19 | 1.26 | 0.17 |
| Q10589 | Isoform 2 of Bone marrow stromal antigen 2 | BST2 | 1.80 | 0.88 | 1.97 | 0.30 | -0.33 | 0.63 | -0.12 | 0.60 |

**Supplementary Figure 1**

**
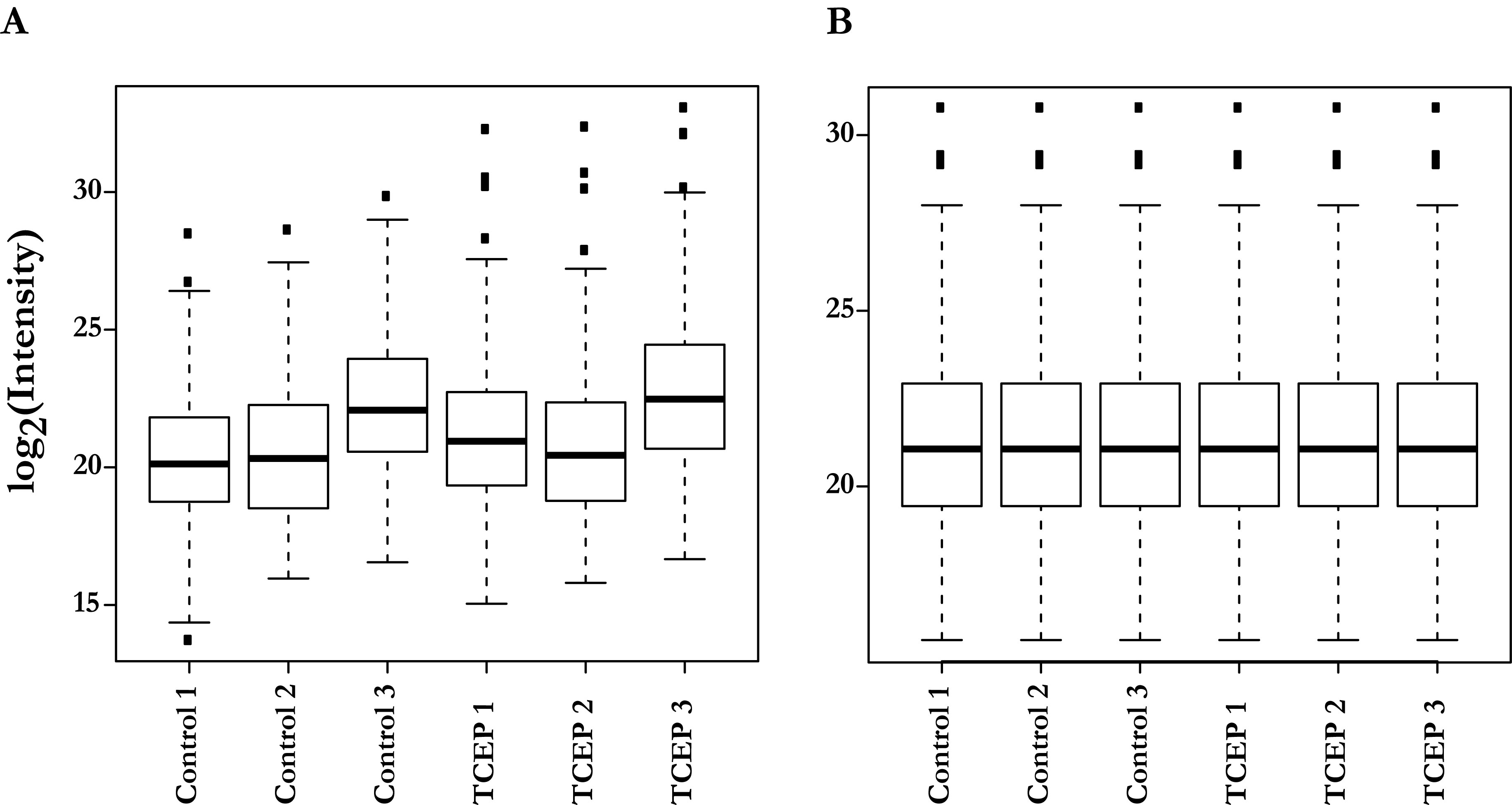
**

**Normalisation of SH-IQ mass spectrometry data: Box and whisker plots.**

The median protein intensity of each experiment is shown as a thick black line surrounded by a box representing the first to third quartile range. The whiskers that extend at both sides of the box indicate the 1.5x interquartile range (IQR) from the first and third quartile, respectively. Outliers that are more than 3x IQR below the first quartile or above the third quartile are shown as closed black circles. The distribution of protein intensity before (A) and after (B) quantile normalisation shows the differences in median intensities can be successfully normalised between experiments (1-3) and samples (Control and TCEP-reduced 2B4 T cells).
